# DNA methylation analysis in patients with neurodevelopmental disorders improves variant interpretation and reveals complexity

**DOI:** 10.1016/j.xhgg.2024.100309

**Published:** 2024-05-15

**Authors:** Slavica Trajkova, Jennifer Kerkhof, Matteo Rossi Sebastiano, Lisa Pavinato, Enza Ferrero, Chiara Giovenino, Diana Carli, Eleonora Di Gregorio, Roberta Marinoni, Giorgia Mandrile, Flavia Palermo, Silvia Carestiato, Simona Cardaropoli, Verdiana Pullano, Antonina Rinninella, Elisa Giorgio, Tommaso Pippucci, Paola Dimartino, Jessica Rzasa, Kathleen Rooney, Haley McConkey, Aleksandar Petlichkovski, Barbara Pasini, Elena Sukarova-Angelovska, Christopher M. Campbell, Kay Metcalfe, Sarah Jenkinson, Siddharth Banka, Alessandro Mussa, Giovanni Battista Ferrero, Bekim Sadikovic, Alfredo Brusco

**Affiliations:** 1 Department of Medical Sciences, University of Turin, 10126 Turin, Italy; 2 Verspeeten Clinical Genome Centre, London Health Sciences Centre, London, ON N6A5W9, Canada; 3 Department of Pathology and Laboratory Medicine, Western University, London, ON N6A3K7, Canada; 4 Molecular Biotechnology Center “Guido Tarone” University of Turin, 10126 Turin, Italy; 5 Department of Molecular Biotechnology and Health Sciences, University of Turin, CASSMedChem, 10126 Turin, Italy; 6 Medical Genetics Unit, Città della Salute e della Scienza University Hospital, 10126 Turin, Italy; 7 Medical Genetics Unit and Thalassemia Center, San Luigi University Hospital, Orbassano, TO 10049, Italy; 8 Department of Neurosciences Rita Levi-Montalcini, University of Turin, Turin 10126, Italy; 9 Department of Public Health and Pediatric Sciences, University of Turin, 10126 Turin, Italy; 10 Department of Biomedical and Biotechnological Sciences, Medical Genetics, University of Catania, 94124 Catania, Italy; 11 Department of Molecular Medicine, University of Pavia, 27100 Pavia, Italy; 12 Neurogenetics Research Center, IRCCS Mondino Foundation, 27100 Pavia, Italy; 13 IRCCS Azienda Ospedaliero-Universitaria di Bologna, 40138 Bologna, Italy; 14 Department of Immunology and Human Genetics, Faculty of Medicine, University "Sv. Kiril I Metodij", Skopje 1000, Republic of Macedonia; 15 Department of Endocrinology and Genetics, Faculty of Medicine, University “Sv. Kiril I Metodij”, Skopje 1000, Republic of Macedonia; 16 Manchester Centre for Genomic Medicine, St. Mary’s Hospital, Manchester University NHS Foundation Trust, Health Innovation Manchester, Manchester M13 9WL, UK; 17 Division of Evolution, Infection & Genomics, Faculty of Biology, Medicine and Health, The University of Manchester, Manchester M13 9WL, UK; 18 Pediatric Clinical Genetics Unit, Regina Margherita Childrens' Hospital, 10126 Turin, Italy; 19 Department of Clinical and Biological Sciences, University of Turin, 10043 Turin, Italy

**Keywords:** episignatures, neurodevelopmental disorders, BAFopathy, Coffin-Siris syndrome, DNA methylation, SMARCA2, SMARCA4, SMARCB1, ARID1A, ARID1B

## Abstract

Analysis of genomic DNA methylation by generating epigenetic signature profiles (episignatures) is increasingly being implemented in genetic diagnosis. Here we report our experience using episignature analysis to resolve both uncomplicated and complex cases of neurodevelopmental disorders (NDDs). We analyzed 97 NDDs divided into (1) a validation cohort of 59 patients with likely pathogenic/pathogenic variants characterized by a known episignature and (2) a test cohort of 38 patients harboring variants of unknown significance or unidentified variants. The expected episignature was obtained in most cases with likely pathogenic/pathogenic variants (53/59 [90%]), a revealing exception being the overlapping profile of two *SMARCB1* pathogenic variants with *ARID1A/B*:c.6200, confirmed by the overlapping clinical features. In the test cohort, five cases showed the expected episignature, including (1) novel pathogenic variants in *ARID1B* and *BRWD3*; (2) a deletion in *ATRX* causing MRXFH1 X-linked mental retardation; and (3) confirmed the clinical diagnosis of Cornelia de Lange (CdL) syndrome in mutation-negative CdL patients. Episignatures analysis of the in BAF complex components revealed novel functional protein interactions and common episignatures affecting homologous residues in highly conserved paralogous proteins (SMARCA2 M856V and SMARCA4 M866V). Finally, we also found sex-dependent episignatures in X-linked disorders. Implementation of episignature profiling is still in its early days, but with increasing utilization comes increasing awareness of the capacity of this methodology to help resolve the complex challenges of genetic diagnoses.

## Graphical abstract

**Figure:**
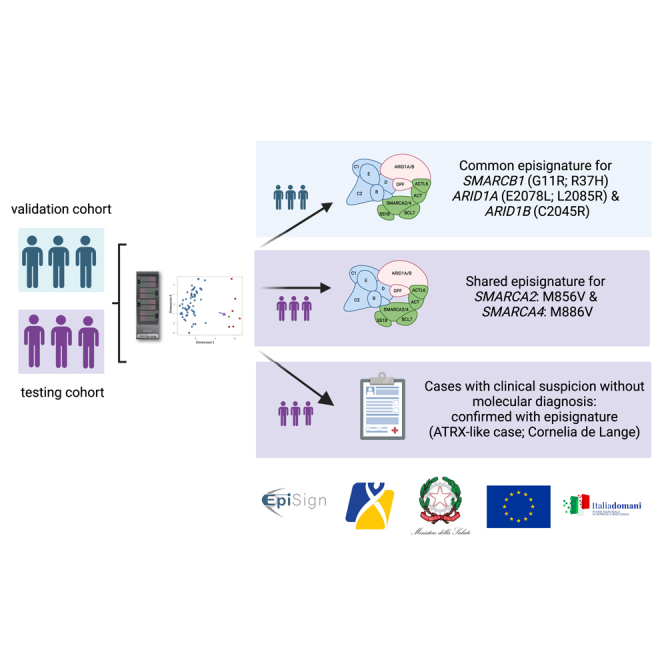

## Teaser

Episignature analysis of DNAm is shown here to be a valuable diagnostic aid in individuals with NDDs that carry genetic variants of uncertain significance. Unexpectedly, this method also resolved complex NDD cases revealing variant-specific episignatures in genes of the BAF complex.

## Introduction

Neurodevelopmental disorders (NDDs) are a group of heterogeneous childhood conditions that include developmental delay, intellectual disability, language delay, and epilepsy. These disorders are characterized by an underlying heritable component affecting different genes whose products are often part of complex pathways required for different stages of embryonic neurodevelopment. Alongside their genetic heterogeneity, NDDs are characterized by broad phenotypic diversity in their clinical presentation, which is the major confounding factor when trying to establish genotype-phenotype correlations.^1^

Both technical advances and cost reductions have allowed chromosomal microarrays (CMAs) and exome sequencing (ES) to emerge as the tier 1 genomic applications for NDD diagnostics. These methods are now widely used and recommended in clinical practice.^2^^,^^3^^,^^4^ Although often successful in detecting underlying genetic causes, a large proportion of cases remain unsolved using these methods. Several factors that can negatively affect the detection rate of causative variants include technical limitations, such as focusing solely on analyzing coding sequences or potentially overlooking insertions or deletions or small exon deletions. A further explanation resides in our inability to establish a causal relationship between a change in DNA sequence and the clinical presentation of the patient. Such DNA changes are classified as variants of uncertain clinical significance (VUS). Among the reasons for classifying a variant as a VUS are (1) the patient’s phenotype does not entirely correspond with the known phenotypes associated with the gene in question; (2) family segregation analyses are missing; or (3) functional assays that prove the causative role of a variant are unavailable. In these patients, a paradigm change has led to the development of new diagnostic tools that are no longer based on modifications in the genome, but based on studying changes in the methylation status of the genome, or epigenome.

Changes in DNA or histone methylation have been identified in a variety of human diseases and, more relevantly for us, in patients with NDDs.^5^^,^^6^ Indeed, numerous NDDs have been categorized as chromatinopathies, caused by variants in genes encoding proteins that are part of the epigenetic methylating machinery. These proteins function variously as writers, erasers, readers, or remodelers of chemical chromatin marks.^7^ Malfunction of these proteins is expected to have various downstream epigenetic consequences. These consequences include subtle changes in DNA methylation (DNAm) across the genome; these changes occur early in embryonic development in numerous tissues, including cells of peripheral blood.^6^^,^^8^

An expanding number of chromatinopathies have been shown to have unique genomic DNAm patterns named epigenetic signatures, or episignatures.^9^ As highly sensitive and specific biomarkers, these episignatures represent a quick and specific assay for a particular gene involved in NDD pathogenesis, and can be applied to classify variants of dubious clinical significance. Currently, more than 65 rare disorders exhibit a distinctive genome-wide DNAm profile when analyzed with the EpiSign v.3 clinical methylation assay.^10^ As the data from EpiSign assays accumulate, novel features of episignatures are starting to emerge. For example, (1) we now know that variants in genes which do not encode for chromatin-related genes can also present distinctive episignatures^11^^,^^12^; (2) the same episignature may be exhibited by variants in genes which encode for multi-protein complexes, as is the case of the so-called BAFopathies, which affect the components of BAF protein complex^13^; (3) the same gene may exhibit different episignatures, depending on the protein domain where the variant is located, as in the complex NDD Helsmoortel-Van der Aa syndrome (OMIM: 615873)^14^; and (4) even single amino acid changes can have a distinct episignature (SMARCA4 M886V).^10^ Finally, copy number variants (CNVs) associated with a genomic disorder can also show distinct DNAm patterns.^15^^,^^16^

In this report, we describe our experience of using the EpiSign assay and episignature analysis with a study cohort of 97 patients with NDDs.

## Material and methods

### Study cohort

Our study group comprised 97 unrelated patients with NDDs selected from a large project focused on genetic screening of NDD cases (NeuroWES). Patients were evaluated by an experienced pediatrician and/or clinical geneticist who provided the phenotype and, when needed, reverse phenotyping. The patients were divided into three categories (see Tables 1 and S1): (1a) validation cohort #1a, which consisted of 34 NDD cases with pathogenic or likely pathogenic single nucleotide variants (SNVs) in a gene with known disease-specific methylation patterns or episignatures that are listed in the EpiSign v.3 classifier; (1b) validation cohort #1b, which consisted of 25 NDD cases with pathogenic or likely pathogenic CNVs that are also listed in the EpiSign v.3 classifier; (2) an uncertain cohort composed of 18 NDD cases with an SNV/CNV VUS or with a strong clinical suspicion but no specific variant identified, and (3) 20 unresolved NDD cases defined by females or mothers of unresolved male cases that showed skewed X chromosome inactivation (XCI) of more than 80% (Supplemental Materials and methods).^17^

**Table 1:** Cases tested using EpiSign v.3 classifier

	Sample ID	Sex	Gene	Variant	ACMG/AMP	Phenotype/diagnosis^a^	Epi V4 result	Notes
**1a) Validation cohort: SNVs in genes with known episignatures (34 cases)**								

1	NWM-030D	F	*ADNP*	NM_001282531.3: c.539_542del p.(Val180fs)	P	HVDAS	HVDAS_T	*ADNP* C-term sign.
2	GM223306	F	*ADNP*	NM_001282531.3: c.2454C>G p.(Tyr818Ter)	P	HVDAS	HVDAS_T	*ADNP* C-term sign.
3	121623	M	*ANKRD11*	NM_013275.6: c.439C>T p.(Gln147Ter)	P	KBGS	KBGS	
4	BA2012002	F	*ANKRD11*	NM_013275.6: c.211_226+1del p.?	P	KBGS	KBGS	
5	NWM-218D	M	*ANKRD11*	NM_013275.6: c.1903_1907del p.(Lys635fs)	P	KBGS	KBGS	
6	NMW-035D	M	*ARID1A*	NM_006015.6: c.6232G>A p.(Glu2078Lys)	LP	CSS2	CSS_c.6200	subregion episignature
7	160759	F	*ARID1B*	NM_001374828.1: c.5825G>A p.(Trp1942Ter)	LP	CSS1	BAFopathy	broad BAFophaty epis.
8	142220	M	*CHD7*	NM_017780: c.3082A>G p.(Ile1028Val)	LP	CHARGE	CHARGE	
9	FS0208013	M	*CHD7*	NM_017780: c.6194G>A p.(Arg2065His)	LP	CHARGE	CHARGE	
10	110562	M	*CHD8*	NM_001170629.2: c.2025-1G>C p.?	LP	IDDAM	IDDAM	
11	110212	M	*CREBBP*	NM_004380.3:c.3779 + 1G>A p.?	P	RSTS1	RSTS	broad RSTS epis.
12	141444	M	*EHMT1*	NM_024757.5: c.3331T>A p.(Cys1111Ser)	P	KLEFS1	KLEFS	broad KLEFS epis.
13	131361	M	*EHMT1*	NM_024757.5: c.3001del p.(Asp1001fs)	P	KLEFS1	KLEFS	broad KLEFS epis.
14	GM181933	M	*EHMT1*	NM_024757.5: c.508del p.(Gln170fs)	P	KLEFS1	KLEFS	broad KLEFS epis.
15	GM184039	F	*EP300*	NM_001429.4: c.3671 + 5G>C p.?	LP	RSTS2	RSTS1	**discordant**
16	NWM-019D	M	*EZH2*	NM_004456.5: c.2015T>G p.(Phe672Cys)	LP	WVS	PRC2	
17	NWM-088D	F	*HIST1H1E*	NM_005321.3: c.458_460del p.(Lys152fs)	P	RMNS	RMNS	
18	GM201880	F	*KAT6A*	NM_006766.5: c.2927del p.(Gly976Valfs)	P	ARTHS	ARTHS	
19.1	121116	M	*KDM5C*	NM_004187.5: c.1204G>A p.(Asp402Asn)	LP	MRXSCJ	MRXSCJ	**discordant**
19.2	121886	F	*KDM5C*	NM_004187.5: c.1204G>A p.(Asp402Asn)	LP	MRXSCJ		
19.3	121888	F	*KDM5C*	NM_004187.5: c.1204G>A p.(Asp402Asn)	LP	MRXSCJ	**negative**	**discordant**
20	NWM-192D	F	*KMT2A*	NM_001197104.2: c.4777del p.(Arg1593fs)	P	WDSTS	WDSTS	
21	GM194228	M	*KMT2D*	NM_003482.4: c.4395dup p.(Lys1466fs)	P	KABUK1	Kabuki	
22	NWM-031D	F	*KMT2D*	NM_003482.4: c.13795_13802del p.(Ala4599fs)	P	KABUK1	Kabuki	
23	NWM-024D	F	*PHF6*	NM_001015877.2: c.890G>T p.(Cys297Phe)	LP	BFLS	**negative**	**discordant**
24.1	NWM-163D1	M	*PQBP1*	NM_001032383.2: c.457_459del p.(Arg153fs)	P	RENS1	RENS1	
24.2	NWM-163D2	M	*PQBP1*	NM_001032383.2: c.457_459del p.(Arg153fs)	P	RENS1	RENS1	
25	GM182051	M	*PQBP1*	NM_001032383.2: c.233C>A p.(Pro78Gln)	LP	RENS1	RENS1	
26	GM173348	F	*SETD1B*	NM_001353345.2: c.598_600del p.(Gln200fs)	P	IDDSELD	IDDSELD	
27	GM223349	M	*SETD5*	NM_001080517.3: c.868_872del p.(Arg290fs)	P	MRD23	MRD23	
28	GM223350	F	*SETD5*	NM_001080517.3: c.3848_3849insC p.(Ser1286fs)	P	MRD23	MRD23	
29	GM190941	M	*SMARCA4*	NM_003072.5: c.3068A>G p.(Glu1023Gly)	LP	CSS4	**negative**	**discordant**
30	GM223379	F	*SMARCA4*	NM_003072.5: c.1646G>T p.(Arg549Leu)	LP	CSS4	**negative**	**discordant**
31	GM223380	F	*SMARCB1*	NM_003073.5: c.110G>A p.(Arg37His)	LP	CSS3	CSS_c.6200	**discordant**
32	GM183514	F	*SMC1A*	NM_006306.4: c.1276_1282del p.(Arg426fs)	LP	CDLS2	CDLS	broad CDLS epis.
33	130091	M	*SOX11*	NM_006306.4: c.159G>T p.(Met53Ile)	P	CSS9	CSS9	
34	131749	M	*SRCAP*	NM_006662.3: c.7937_7938del p.(Val2646fs)	P	FLHS	FLHS	

**1b) Validation cohort: CNVs with known EpiSignatures (25 cases)**								

1	NWM-020D	F	*SETD5*	3p25.3(9091710–12334937)x1	P	MRD23	MRD23	
2	162391	M	*SETD5*	3p26.3(52266–10683525)x1	P	MRD23	MRD23	
3	GM190395	F	*4p16.13del*	4p16.13(71660–6479683)x1	P	WHS	WHS	
4	GM200157	F	*4p16.13del*	4p16.13(71660–13395123)x1	P	WHS	WHS	
5	T223	M	*5q35del*	5q35(176463495–177956831)x1	P	SOTOS	Sotos	
6	S288	M	*5q35dup*	5q35(176412680–177477797)x3	P	HMA	HMA	
7	GM201583	F	*7q11.23del*	7q11.23(73312582–74924037)x1	P	WBS	WBS	
8	GM192375	M	*7q11.23del*	7q11.23(73312582–74725057)x1	P	WBS	WBS	
9	GM193789	F	*7q11.23dup*	7q11.23(73312582–74725057)x3	P	WBS dup	WBS dup	
10	111884	F	*EHMT1*	9q34.3(136428708–138059695)x1	P	KLEFS1	KLEFS	broad KLEFS epis.
11	131568	F	*EHMT1*	9q34.3(137447506–137984409)x1	P	KLEFS1	KLEFS	broad KLEFS epis.
12	161978	M	*EHMT1*	9q34.3(135866376–138114463)x1	P	KLEFS1	KLEFS	broad KLEFS epis.
13	GM181473	F	*EHMT1*	9q34.3(137666340–138059695)x1	P	KLEFS1	KLEFS	broad KLEFS epis.
14	N821	F	*CREBBP*	16p13.3(3461539–3805666)x1	P	RSTS1?	RSTS1	
15	112066	M	*22q11.21del*	22q11.21(18932429–21086225)x1	P	VCFS/DGS	VCFS/DGS	22q11.21DS LCR A-D
16	112408	M	*22q11.21del*	22q11.21(18932429–21086225)x1	P	VCFS/DGS	VCFS/DGS	22q11.21DS LCR A-D
17	141583	M	*22q11.21del*	22q11.21(18932429–21086225)x1	P	VCFS/DGS	VCFS/DGS	22q11.21DS LCR A-D
18	160892	M	*22q11.21del*	22q11.21(18932429–21086225)x1	P	VCFS/DGS	VCFS/DGS	22q11.21DS LCR A-D
19	161876	F	*22q11.21del*	22q11.21(18932429–21086225)x1	P	VCFS/DGS	VCFS/DGS	22q11.21DS LCR A-D
20	GM192617	F	*22q11.21del*	22q11.21(18932429–21086225)x1	P	VCFS/DGS	VCFS/DGS	22q11.21DS LCR A-D
21	150284	M	*22q11.21del*	22q11.21(18932429–20324240)x1	P	VCFS/DGS	VCFS/DGS	22q11.21DS LCR A-B
22	162620	M	*22q11.21del*	22q11.21(18932429–20324240)x1	P	VCFS/DGS	VCFS/DGS	22q11.21DS LCR A-B
23	142071	F	*17q21.3del*	17q21.3(45640337–46082496)x1	P	KDVS	KDVS	
24	152118	F	*17q21.3del*	17q21.3(45640337–46082496)x1	P	KDVS	KDVS	
25	GM181681	F	*17q21.3del*	17q21.3(45640337–46267672)x1	P	KDVS	KDVS	

**2) Confirmation of pathogenicity in cases with VUS (SNV/CNV) or clinical suspicion without any variant found (18)**								

1	160708	M	*ARID1B*	NM_001374828.1: c.2480C>T p.(Ala827Val)	VUS	CSS1	BAFopathy	broad BAFophaty epis.
2	150163	M	*ARID1B*	NM_001374828.1: c.3589G>A p.(Asp1197Asn)	VUS	CSS1	CdLS	new diagnosis suggested
3	NWM-116D	M	*BRWD3*	NM_153252.5: c.1233-7_1233-3del p.?	VUS	MRX93	MRX93	VUS -> LP
4	GM173400	F	*SMARCA2*	NM_003070.5: c.2566A>G p.(Met856Val)	VUS	NCBRS	BIS	**discordant**
5	GM203135	F	*KMT2A*	NM_001197104.2: c.5959G>A p.(Glu1987Lys)	VUS	NDD	negative	VUS -> LB
6.1	140556	M	*SMARCA2*	NM_003070.5: c.2296C>G p.(Leu766Val)	VUS	NCBRS	negative	VUS -> LB
6.2	140558	M	*SMARCA2*	NM_003070.5: c.2296C>G p.(Leu766Val)	VUS	NCBRS	negative	VUS -> LB
7	NWM-236D	F	*NIPBL*	No variant identified	–	CdLS	CdLS	new diagnosis suggested
8	S890	M	*22q11.21del*	22q11.21(20379137–21151128)x1	VUS	VCFS/DGS	VCFS/DGS	22q11.21DS LCR B-D
9	GM203534	F	*22q11.21del*	22q11.21(20400132–21086225)x1	VUS	VCFS/DGS	negative	22q11.21DS LCR B-D
10	140901	F	*22q11.21del*	22q11.21(20400132–21086225)x1	VUS	VCFS/DGS	Negative	22q11.21DS LCR B-D
11	R641	M	*22q11.21del*	22q11.21(21444416–22574173)x1	VUS	VCFS/DGS	negative	22q11.21DS LCR B-D
12	141494	F	*22q11.21del*	22q11.21(21444416–22574173)x1	VUS	VCFS/DGS	negative	22q11.21DS LCR B-D
13	S257	F	*22q11.21del*	22q11.21(20721287–21025669)x1	VUS	VCFS/DGS	negative	
14	131777	M	*22q11.21del*	22q11.22(21968733–22215491)x1	VUS	VCFS/DGS	negative	
15	GM194370	M	*22q11.21del*	22q11.22(21968733–22215491)x1	VUS	VCFS/DGS	negative	
16	GM193223	M	*22q11.21del*	22q11.22(21968733–22215491)x1	VUS	VCFS/DGS	negative	
17	GM191544	M	*22q11.21del*	22q11.22(22655814–23285204)x1	VUS	VCFS/DGS	negative	
18	GM193550	M	*22q11.21del*	22q11.22(22655814–23285204)x1	VUS	VCFS/DGS	negative	

**3) Cases with skewed XCI (20)**								

1	NWM-021D	F		No variant identified; sk.-XCI (97%)		*NDD*	MRD23/KBGS	new diagnosis suggested
2	141078	M		No variant identified; sk.-XCI (92%)		*NDD*		
3	162199	M		No variant identified; sk.-XCI (91%)		*NDD*		
4	150692	M		No variant identified; sk.-XCI (95%)		*NDD*		
5	140041	M		No variant identified; sk.-XCI (90%)		*NDD*		
6	160035	M		No variant identified; sk.-XCI (88%)		*NDD*		
7	152994	F		No variant identified; sk.-XCI (93%)		*NDD*		
8	141345	F		No variant identified; sk.-XCI (94%)		*NDD*		
9	GM210581	F		No variant identified; sk.-XCI (100%)		*NDD*		
10	150689	F		No variant identified; sk.-XCI (55%)^b^		*NDD*		
11	GM170809	F		No variant identified; sk.-XCI (84%)		*NDD*		
12	29D	F		No variant identified; sk.-XCI (97%)		*NDD*		
13	6D	F		No variant identified; sk.-XCI (95%)		*NDD*		
14	173D	F		No variant identified; sk.-XCI (93%)		*NDD*		
15	164D	M		No variant identified; sk.-XCI (96%)		*NDD*		
16	FM0-711016_92	M		No variant identified; sk.-XCI (100%)		*NDD*		
17	90D	M		No variant identified; sk.-XCI (94%)		*NDD*		
18	43D	M		No variant identified; sk.-XCI (91%)		*NDD*		
19	22D	M		No variant identified; sk.-XCI (91%)		*NDD*		
20	111092	M	*ATRX*	*ATRX* exon 3–4 deletion; sk.-XCI (100%)	P	MRXFH1	MRXFH1	case solved by episign.

ARTHS, Arboleda-Tham syndrome (OMIM: 616268); CDLS1, Cornelia de Lange syndrome-1 (OMIM:122470); CDLS2, Cornelia de Lange syndrome-2 (OMIM: 300590); CSS1, Coffin-Siris syndrome-1 (OMIM: 135900); CSS2, Coffin-Siris syndrome-2 (OMIM: 614607); CSS3, Coffin-Siris syndrome-3 (OMIM: 614608); CSS4, Coffin-Siris syndrome-4 (OMIM: 614609); CSS9, Coffin-Siris syndrome-9 (OMIM: 615866); HMA, Hunter-McAlpine (OMIM 601379); HVDAS, Helsmoortel-Van der Aa syndrome (OMIM: 615873); IDDAM, intellectual developmental disorder with autism and macrocephaly (OMIM: 615032); IDDSELD, intellectual developmental disorder with seizures and language delay (OMIM: 619000); KABUK1, Kabuki syndrome-1 (OMIM: 147920); KBGS, KBG syndrome (OMIM: 148050); KDVS, Koolen-De Vries syndrome (OMIM: 610443); KLEFS1, Kleefstra syndrome-1 (OMIM: 610253); MRD23, autosomal dominant intellectual developmental disorder-23 (OMIM: 615761); MRXSCJ, Claes-Jensen type of X-linked syndromic intellectual developmental disorder (OMIM: 300534); MRX93-Mental retardation X-linked 93 CHARGE (OMIM: 214800); MRXFH1, X-linked intellectual disability-hypotonic facies syndrome-1 (OMIM: 309580); NDD, neurodevelopmental disorder; RENS1, Renpenning syndrome (OMIM: 309500); RMNS, Rahman syndrome (OMIM: 617537); sk-XCI, skewed XCI >80%; SOTOS, Sotos syndrome (OMIM: 117550); WBS, Williams-Beuren syndrome (OMIM: 194050); WBS dup, duplication of genes lying within the critical region for Williams-Beuren syndrome (OMIM: 609757); WDSTS, Wiedemann-Steiner syndrome (OMIM: 605130); WHS, Wolf-Hirschhorn syndrome (OMIM: 194190); WVS, Weaver syndrome (OMIM: 277590); X-linked intellectual developmental disorder-93 (OMIM: 300659). Numbering of patients: 19.1, 19.2, 19.3, 24.1, 24.2, 6.1, and 6.2 refers to siblings carrying the same genetic variant.

a The phenotype indicated is the clinical diagnosis of the reported case. A question mark indicates a suspected diagnosis.

b The proband's mother (case 150691) was 95% skewed.

All SNVs were confirmed by Sanger sequencing; both SNVs and CNVs were classified according to the American College of Medical Genetics and Genomics (ACMG)/Association for Molecular Pathology (AMP) guidelines.^18^^,^^19^^,^^20^ Genomic sequencing for case 150163 is reported in Supplemental Materials and methods.

### Sample and microarray processing

DNAm array data were performed using MethylationEPIC BeadChip array (EPIC array) at the Verspeeten Clinical Genome Center, London Health Sciences Center in London, Canada, following the manufacturer’s protocols and analyzed at the same center, as previously described.^10^^,^^21^^,^^22^ Methylation data for each sample were compared with all 57 DNAm profiles (associated with 65 genetic syndromes) included in the EpiSign v.3 classifier.

### DNAm analysis by EpiSign

The DNAm data for each sample was compared to the Episign Knowledge Databases (EKDs) using the support vector machine (SVM)-based classification algorithm as previously described.^10^^,^^21^^,^^22^ The EKD includes thousands of clinical peripheral blood DNAm profiles from disorder-specific reference and normal controls (general population samples with various age and racial backgrounds). The SVM decision values were converted to methylation variant pathogenicity (MVP) scores ranging from 0 to 1, using the Platt scaling method. MVP scores indicate the prediction confidence for the specific episignature. Scores of greater than 0.01 undergo a secondary review using hierarchical and multidimensional scaling (MDS) clustering plots associated with the episignature. The final EpiSign result is a combination of the three assessed parameters: MVP scores, hierarchical plots, and MDS plots. The result is reported with a confidence level relative to the reference episignature cohorts, where high confidence indicates agreement among all three parameters and moderate confidence indicates disagreement in at least one of the three parameters.

### Three-dimensional protein modeling

The BAF complex model was constructed by selecting suitable experimental structures to be used as scaffold and by superposing the corresponding human proteins in their full-length version as found in the Alpha Fold Database. Specifically, the full BAF complex was constructed based on the work of He and co-workers.^23^ The structure used as template was resolved with cryo electron microscopy; the PDB code is 6LTJ, the resolution is 3.70 Å. The AlphaFold structures for the human full-length SMARCB1 and ARID1A/B were superposed (UniProt accession Nos. Q12824, O14497, and Q8NFD5). The BAF base module originated from the PDB structure 6LTH (cryo electron microscopy, human, resolution 3.00 Å).

All protein structure manipulations were performed with the Molecular Operating Environment (version 2022), from ChemComp (www.chemcomp.com) by first employing the structure preparation pipeline with standard settings. Then, PDB templates and AlphaFold models were imported in the same session and superposed with the check and realign procedure. Finally, hydrogens were added and partial charges assigned according to the parameters of the AMBER 10:EHT forcefield.^24^ Then the overall structure was inspected for clashes after removing the original chains in the PDB template. Clashes were avoided with multiple local minimization cycles and a final global minimization was performed, obtaining models hosting full-length SMARCB1 and ARID1A/B chains. The procedure was obtained for the wild-type complex or by introducing the selected mutations with the MOE protein builder tool (www.chemcomp.com). Before global minimization, sidechain optimization for the mutant residue was performed. The interaction energy between SMARCB1 and ARID1A/B in the complex was estimated through the MOE energy tool and considered the sum of all terms. The same procedure for chain superposition was obtained when comparing the structures of SMARCA2 and SMARCA4 (UniProt references P51531 and P51532, respectively). The sequence alignment was based on the BLOSUM62 matrix and the structural component considered alpha carbons. All other settings of the superposition were default.

For the determination of newly formed interactions, standard MOE cutoffs were considered and the choice of relevant atoms to display relative distance was made upon visual inspection.

### Ethics approval and consent to participate

All individuals and families from the different institutions agreed to participate in this study and signed appropriate consent forms according to the Declaration of Helsinki [Ethics Committee of University of Turin (n. 0060884) and University of Skopje (n. 03–6116/7)]. Consent for publication has been obtained from individuals or their parent or legal guardian in case of children, whose clinical details or images are reported.

## Results

### Characteristics of the cohorts used for episignature analysis

This study involving episignature analysis is based on a cohort of 97 unrelated patients with NDDs (Table 1), divided into the following groups: (1a) the SNV validation cohort, which analyzed DNA samples from 34 cases with likely pathogenic or pathogenic SNVs in disease-associated genes with an established diagnostic EpiSign methylation profile; (1b) the CNV validation cohort, which analyzed DNA samples from 25 cases with likely pathogenic or pathogenic CNVs involving 26 different genes/CNVs with an established diagnostic EpiSign methylation profile^10^; (2) the VUS/undetected variant cohort, which consisted of 18 samples from patients with either a VUS (SNV or CNV) or with a clinically suspected NDD but no variant detected by preceding genome analyses; (3) the skewed XCI cohort, which consisted of 20 samples with a clinical diagnosis of NDD, without a causative X-linked variant identified by exome analysis.

### EpiSign analysis of the SNV and CNV control cohorts

The combined SNV validation cohort (34 samples) and the CNV validation cohort (25 samples) represented our 59 control samples where the EpiSign profile expected of the SNV/CNV is known *a priori*.^10^ In fact, in 53 of the 59 specimens analyzed (28/34 SNVs; 25/25 CNVs), the methylation pattern obtained correctly matched the established EpiSign profile, identifying the correct episignatures that were gene/CNV-specific, protein domain-specific (e.g., *ADNP* central nonsense variants in Helsmoortel-Van der Aa syndrome) or protein complex-specific (e.g., pertaining to the BAFopathies, Cornelia De Lange syndrome [CdLS]), or Kabuki syndrome) (Table 1).

Discordant results were obtained in 6 of 34 SNV validation cohort samples. Of these, three did not match the expected episignature (Table 1; samples GM184039, 121116, and GM223380) and three did not match any known episignature (Table 1; samples GM190941, GM223379, and NWM-024D). These samples were further investigated to unravel the causes of the discordance.

#### Sample GM184039

The patient (female) is heterozygous for a *de novo* likely pathogenic splicing variant (c.3671+5G>C p.?) in *EP300*, the gene where truncating variants are associated with Rubinstein-Taybi syndrome 2 (RSTS2) (OMIM: 613684). However, the observed methylation profile suggested Rubinstein-Taybi syndrome 1 (RSTS1) (OMIM: 180849), which is associated with the *EP300* partner and paralog, *CREBBP*. This result might suggest that this variant has unexpected effects on the function of the CREBBP/EP300 acetyltransferase complex. In addition, global methylation analysis also revealed hypomethylation at the *GNAS* A/B:TSS-DMR locus, suggestive of pseudohypoparathyroidism, type 1B (PHP1B) (OMIM: 603233). These findings led to a clinical re-evaluation of the patient,^25^ who had slightly increased parathyroid hormone levels and brachydactyly, although other PHP1B-related features (e.g., skeletal, renal, and biochemical abnormalities) were absent. Additional studies are ongoing to confirm the role of the *EP300* variant and the possibility that the distinctive methylation profile may be caused by the overlap of these two conditions.

#### Sample 121116

The patient (male) has a hemizygous variant c.1204G>A p.(D402N) in *KDM5C*, a histone demethylase-encoding gene associated with Claes-Jensen syndrome (MRXSCJ) (OMIM: 300534), an X-linked recessive disorder.^17^ The clinical features suggested a milder form of MRXSCJ and the observed episignature was consistent with that of a heterozygous female, supporting this interpretation (Figure S1). We further extended methylation profiling to his sister and mother (cases 19.2 and 19.3) (Table 1), who were carriers of the variant, but without a reported phenotype. In women, the methylation profile was not concordant with MRXSCJ cases; however, the sister clustered with carriers and the mother with controls, although slightly shifted toward heterozygous females (Figure S1). The third discordant sample, GM223380, is discussed with the BAFopathy cases.

#### Samples GM190941 and GM223379

**Figure 1:**
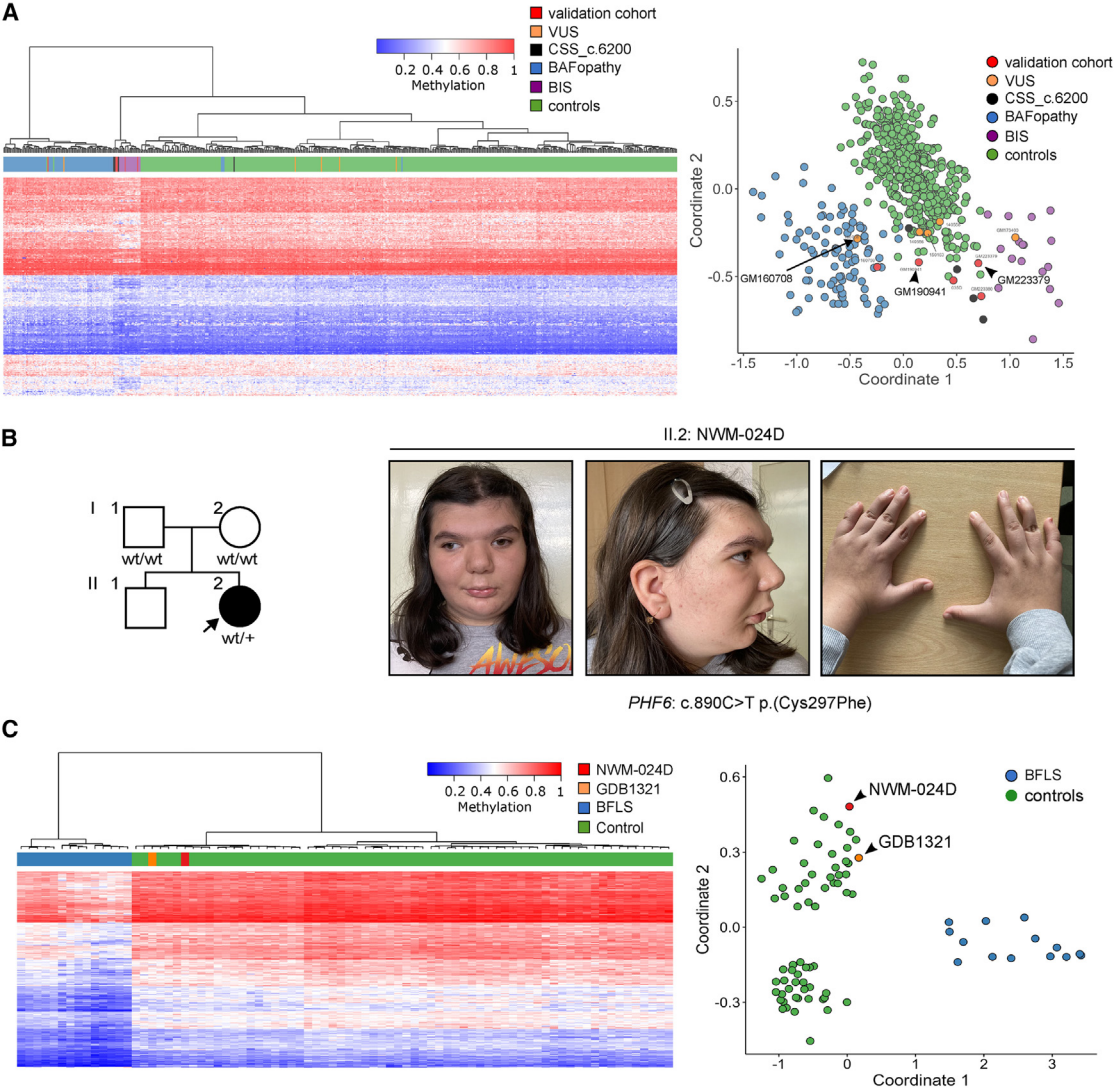
Novel interpretations for discordant episignatures (A) Euclidean hierarchical clustering (heatmap) (left) and MDS plot (right) from two subjects with CSS4 that harbored likely pathogenic variants in *SMARCA4*: GM190941 [c.3068A>G p.(E1023G) and GM223379 (c.1646G>T p.(R549L)]. In the MDS, the DNAm methylation profiles of the CSS4 samples do not cluster with the BAFopathy episignature. The detected episignature is currently undefined and the two patients uncharacterized. Case GM160708 with *ARID1B*:c.2480C>T p.(A827V) had a BAFopathy EpiSign, supporting the diagnosis of a rare case of CSS1 due to a missense variant in *ARID1B*. (B) Family tree of patient NWM-024D (II.2), the second child of healthy parents. She had a *de novo PHF6*:c.890C>T p.(C297F) variant, strongly suggestive of BFLS. Note the coarse and wide face, low-set ears, bitemporal narrowing, hypertelorism, prominent supraorbital ridges, prominent eyebrows, synophrys, long philtrum, carpe-shaped nose, retrognathia, short neck, and brachydactyly (photo at 12 years of age). (C) Left shows the DNAm heatmap of two patients with BFLS, NWM-024D, and GDB1321, the latter being the only other female with BFLS so far analyzed, established BFLS cases and healthy controls. Right, the MDS plot shows clustering of NWM-024D and GDB1321^22^ with controls (green) and not with BFLS cases (blue).

These samples are from two patients that present the clinical features of Coffin-Siris syndrome 4 (CSS4)^26^ and have likely pathogenic variants in the chromatin remodeler *SMARCA4*, a known CSS4-related gene: c.3068A>G p.(E1023G) in GM190941 and c.1646G>T p.(R549L) in GM223379. We expected the methylation profile to fall within the BAFopathy cluster; instead, both cases revealed an episignature that was intermediate between the BAFopathy profile and the profile of blepharophimosis-impaired intellectual disability syndrome (BIS) (OMIM: 619293), an allelic disorder associated with *SMARCA2*, a paralog of *SMARCA4* (Figure 1A). SMARCA4 and SMARCA2 are mutually exclusive catalytic components of the BAF chromatin remodeling complex and their protein sequence alignments show 73% amino acid identity over the whole protein length. Intriguingly, both SMARCA4 variants substitute paralogue-conserved residues: SMARCA4 E1023 corresponds with E993 in SMARCA2, and SMARCA4 R549 to SMARCA R525 (Figure S2). Interestingly the facial dysmorphia of case GM223379 resembled more BIS than CSS4 (narrow palpebral fissures, mild blepharophimosis, epicanthal folds, and ptosis).

#### Sample NWM-024D

The patient (female) has autistic features, global developmental delay, brachy/syndactyly, coarse facial features with strabismus, and was originally described in^17^ (Figure 1B). She is heterozygous for a *de novo* variant c.890G>T p.(C297F) in *PHF6*, the causative gene of Borjeson-Forssman-Lehmann syndrome (BFLS) (OMIM: 301900) (Figure S3). The methylation profile was similar to healthy controls and did not match that of BFLS cases (Figure 1C). Since BFLS is an X-linked recessive disorder, affected cases are males, while heterozygous females are usually unaffected or may present a mild clinical phenotype.^27^ Our proband showed clinical presentation and very similar facial gestalt as the other described female cases carrying a few amino acid distant changes.^27^ The complete X-inactivation skewing was further supporting an X-linked condition. In this case, we are hypothesizing that a sex-related episignature exists for this gene. Indeed, the BFLS EpiSign profile was obtained from male cases of BFLS; the one other female analyzed so far (GDB1321) (Figure 1C)^22^ also showed a methylation pattern similar to controls.

Regarding our analysis of CNVs with known episignatures, our study confirmed that 25 of the 25 CNVs were indeed pathogenic (Table 1). In most cases, these CNVs were associated with contiguous gene syndromes, where a combination of several dosage-sensitive genes causes the disease and likely affects the DNAm pattern. In other cases, the CNV analyzed caused the loss or gain of a single dosage-sensitive gene, revealing a DNAm profile specific for the disease-associated gene in question: e.g., the 5q35 deletion associated with Sotos syndrome involving *NSD1*, the 5q35 duplication associated with Hunter-McAlpine craniosynostosis syndrome (*NSD1*), and the 4p16.13 deletion associated with Wolf-Hirschhorn syndrome (*NSD2*). Among the contiguous gene syndromes, we confirmed two 7q11.23 deletions and one 7q11.23 duplication corresponding with Williams-Beuren syndrome and the reciprocal duplication profiles, respectively. Finally, we confirmed eight cases with the typical 22q11.2DS episignature profile, while the same episignature was excluded in six cases involving variable deletions in the central 22q11.2DS (described below). This confirms previous data suggesting that the 22q11.2DS EpiSign profile is specific for the loss of the 1.5-Mb region known as the DiGeorge syndrome (DGS)/velocardiofacial syndrome (VCFS) critical region.

### EpiSign analysis of the VUS/no variant cohort

In this cohort, we conducted episignature analysis of 18 deeply phenotyped NDD cases with VUS, with the aim of establishing whether or not they were pathogenic. Details of four cases are provided below, where the rest of the cases did not match any of the defined episignature profiles.

#### Samples 160708 and NWM-116D

Sample 160708 was from a patient with CSS1 with a missense VUS c.2480C>T p.(A827V) in *ARID1B*, the known causative gene of CSS1, which encodes a component of the BAF chromatin remodeling complex. The DNAm profile matched the BAFopathy episignature, allowing us to reclassify the variant as likely pathogenic (Figure 1A).

Sample NWM-116D had a maternally inherited variant predicted to affect the acceptor splice site in exon 14 of *BRWD3* [c.1233-7_1233-3del p.?; predicted change −66%] (Figure S4). Other biological samples were unavailable, making it impossible to confirm aberrant splicing by cDNA analysis, but the patient clinically matched the phenotype of MRX93, intellectual developmental disorder, X-Linked 93 (OMIM: 300659) associated with *BRWD3*. The DNAm pattern confirmed this diagnosis, reclassifying the variant as likely pathogenic.

#### Samples 150163 and NWM-236D

**Figure 2:**
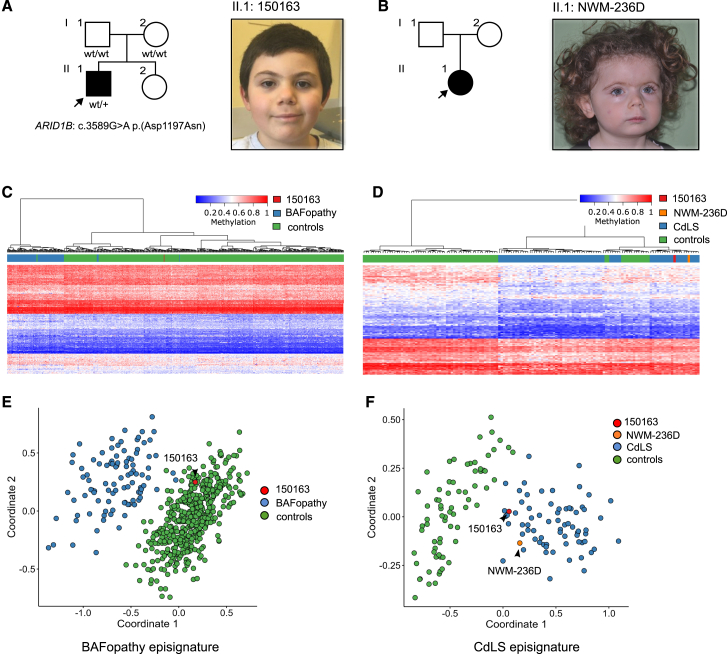
Episignature analysis suggests a diagnosis of CdLS in unsolved cases (A and B) Cases 150163 and NWM-236D of CdLS with no variants identified in CdLS genes by ES/CMA screening. Case 150163 was initially misleading as he had an *ARID1B* c.3220G>A p.(D1074N) *de novo* missense variant. This variant has now been reported in three cases in GnomAD (v.2.1.1), further supporting its likely benign role. (C and D) Heatmaps for cases 150163 (C, left) and NWM-236D (D, right). (E and F) MDS plots for the two patients showed that 150163 did not show a BAFopathy episignature (E, left MDS), whereas both cases clustered with the CdLS profile (F, right MDS).

Sample 150163 was from a patient with a *de novo ARID1B* D1197N VUS (Figure 2A). However, the methylation profile was inconsistent with a BAFopathy and instead compatible with the profile in CdLS (Figure 2B). This result suggested that *ARID1B* D1197N was not pathogenic. Indeed, reverse phenotyping of the patient revealed clinical features suggestive of CdLS (Figure 2A), indicating we may have missed the causative variant in one of the CdLS-associated genes. Further investigation by genome sequencing failed to identify SNVs or structural variants in known CdLS genes (Supplemental materials and methods; Table S2). The CdLS episignature was also identified in NWM-236D, a second patient whose phenotype suggested CdLS but without detectable anomalies by CMA or ES (Figures 2B, 2D, and 2F), again suggesting a missed pathogenic variant in one of the CdLS genes.

Sample GM173400 with a VUS in *SMARCA2* is discussed below. Samples from patients with VCFS/DGS and CNVs of unknown significance are examined separately (see below).

### EpiSign analysis of the skewed XCI cohort

Our last cohort consisted of probands with NDD with no candidate variants after genome analysis but with a family history of skewed XCI, suggesting a disorder with X-linked inheritance.^17^

Among the 20 cases, we found patient NWM-021D had the episignature specific for MRD23_KBG, intellectual developmental disorder, autosomal dominant 23 syndrome (formally mental retardation, autosomal dominant 23 syndrome) and KBG syndrome (Figure S5, bottom), although CMA/ES analyses failed to identify deleterious variants in either of the associated genes, i.e., *SETD5* and *ANKRD11*. Among the possible explanations, there may be a missed variant in these genes or a yet unknown genes associated with this episignature.^10^

#### Sample 111092

The clinical features of this male patient suggested X-linked intellectual disability, hypotonic facies syndrome 1 (MRXFH1) (OMIM: 309580), but no variants were detected by genome analysis. The patient’s mother was also uninformative, however showing completely skewed XCI. The proband’s methylation profile was clearly associated with that of *ATRX*, the causative gene of MRXFH1, which encodes a chromatin remodeler (Figure S5, top). The case was further studied by our group^17^ and finally resolved with the identification of a deletion in *ATRX* of exons 3 and 4 (NM_000489.6: c.134-4884_242+41del p.?).

### Expanding BAFopathy complex episignatures

Genes involved in chromatin remodeling/DNAm are among the most frequently mutated in NDDs,^28^ and episignature analysis of the BAFopathies is rapidly evolving into an opportunity to dissect the function of individual BAF complex proteins at the protein domain, sub-domain down to the single amino acid level. Among the most prominent and most studied BAFopathies are the clinically overlapping syndromes CSS and Nicolaides-Baraitser (NCBRS) (MIM: 601358), caused by variants in BAF complex proteins: *ARID1B* in CSS1, *SMARCB1* in CSS2, and *SMARCA4* in CSS4; and *SMARCA2* in NCBRS. Both syndromes are associated with a broad DNAm episignature, although two sub-episignatures specific for regions or variants in *ARID1A*, *ARID1B* and *SMARCA*4*2* have been reported.^13^^,^^21^^,^^29^ We report here on two cases which add to the current state of the art of the BAFopathies’ episignatures.

#### Samples GM223380 and SMARCB1

**Figure 3:**
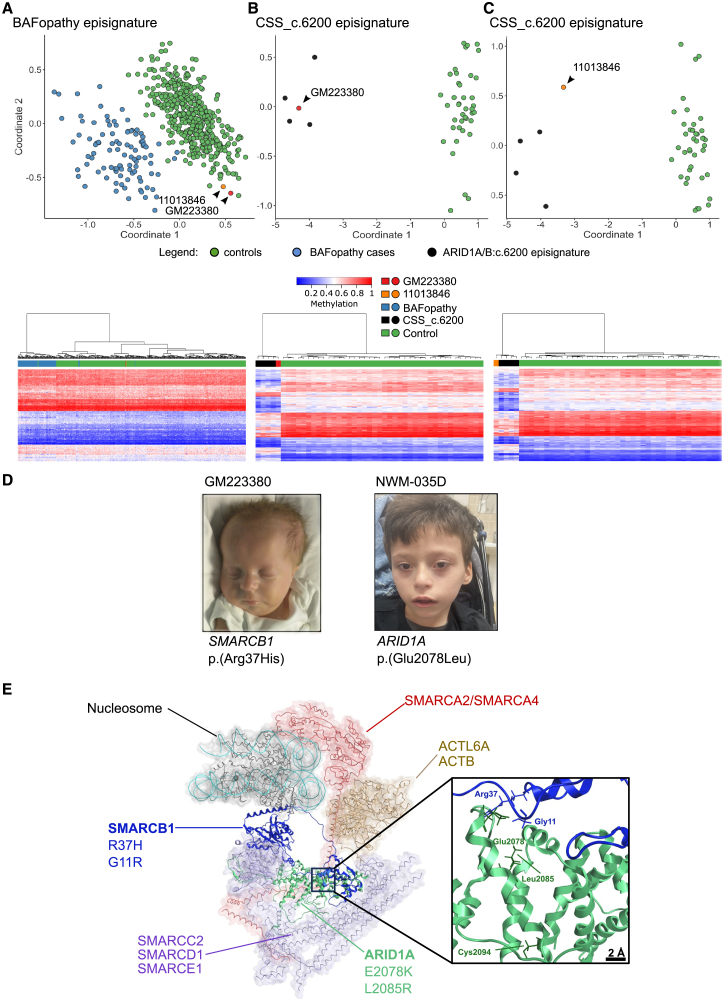
Missense variants in the DNA-binding domain of the SMARCB1 protein reveal a novel rule for the CSS_c.6200 sub-episignature (A–C) MDS plots and heatmaps for two subjects with missense variants in *SMARCB1* [GM223380 c.110G>A p.(R37H) and 11013846 c.31G>A p.(G11R)] show their profiles cluster with cases with the CSS_c.6200 sub-domain episignature, found in individuals with C-terminal variants in *ARID1A* [c.6232G>A p.(E2078K); c.6254T>G p.(L2085R)] and *ARID1B* (c.6133T>C p.(C2045R).^10^. (D) Our two cases with SMARCB1 p.(R37H) and ARID1A p.(E2078L) variants show common facial features with the SMARCB1 p.(R37H) described patients.^30^. (E) Schematic architecture of the human BAFopathy complex. All the variants associated with the CSS_c.6200 sub-domain EpiSign profile encode for amino acids in close spatial proximity of the DNA-binding domain of the SMARCB1 protein where the R37H and G11R reside. This suggests that the CSS_c.6200 sub-domain episignature depends on a specific alteration in BAF complex function.

This sample was part of the SNV control cohort, from a patient with a subtype of CSS3 (OMIM: 614608) carrying the “Kleefstra” variant, characterized by the recurrent *de novo SMARCB1*:c.110G>A p.(R37H) missense substitution. However, the methylation profile did not match the expected broad BAFopathy episignature (Figure 3A), but instead showed a sub-episignature specifically associated with the *ARID1A/B*:c.6200 region identified in cases with missense variants in ARID1A (E2078K, L2085R) or ARID1B (C2045R) (Figure 3B).^10^ The same sub-episignature was observed in a patient with SMARCB1 G11R present in the EKD (Figure 3C).

We compared the clinical features of our SMARCB1 R37H patient with the *ARID1A/B*:c.6200 episignature present in our cohort (NMW-35D; ARID1A E2078K) (Figure 3D). The clinical similarity of these cases was striking, with common features including severe intellectual disability, choroid plexus hyperplasia, hydrocephalus, walking difficulties, and a typical facial gestalt, in line with their common methylation pattern. To explain this pattern, we visually inspected the BAF complex three-dimensional (3D) protein structure, which showed that SMARCB1 R37, SMARCB1 G11, and ARID1A (E2078, L2085)/ARID1B (C2045) were in close spatial proximity (Figure 3E). We also performed a forcefield-based energy estimation of the mutant SMARCB1, ARID1A, and ARID1B proteins. This computational method estimates the global energy of a protein assembly, yielding indications about the strength of intermolecular interactions within the complexes. The results of the interaction energy estimations suggest that the main effect of the mutations is the overall stabilization of the SMARCB1-ARID1A/B complex, owing to the formation of novel intermolecular interactions (Figures S6–S10).

#### Samples GM173400 and SMARCA2

**Figure 4:**
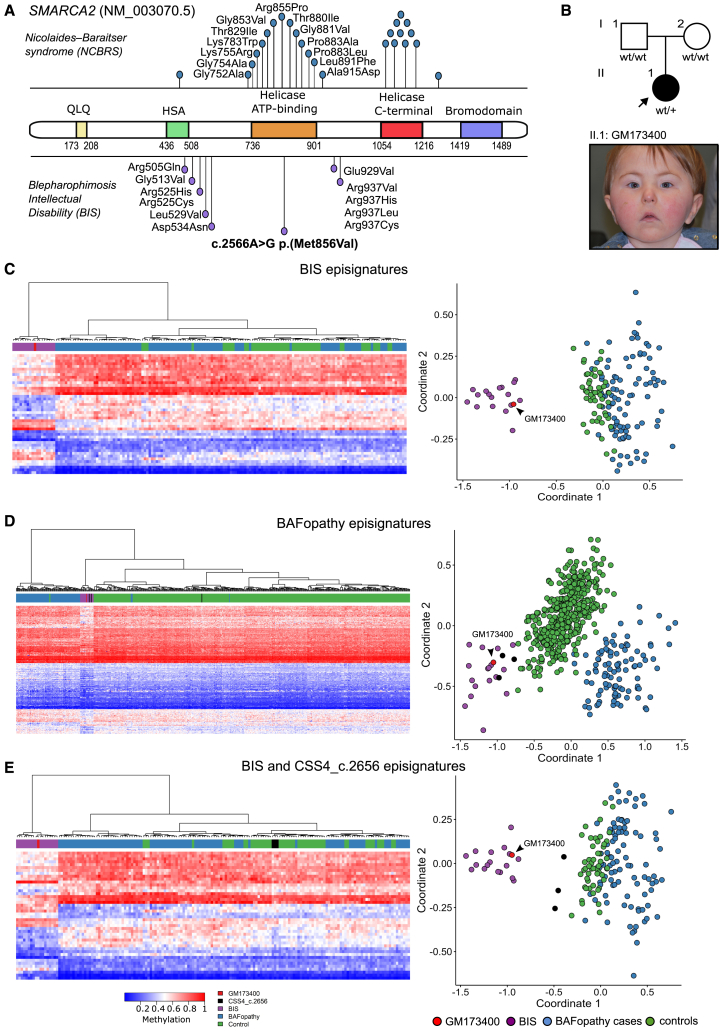
Insights into the distribution of NCBRS/BIS-causative variants (A) Missense variants in *SMARCA2* cause two different syndromes, depending on their location within the protein. The schematic structure of the SMARCA2 protein (figure modified from ref.^29^) shows the five constituent domains with variants associated with NCBRS indicated above the protein and those associated with the BIS below. NCBRS variants cluster in the helicase ATP-binding or helicase C-terminal domain, whereas BIS variants are outside these regions. (B) Pedigree of case GM173400, who is carrier of a *de novo SMARCA2* c.2566A>G p.(M856V) variant. The facial gestalt of GM173400 is compatible with a BIS phenotype. (C–E) Euclidean hierarchical clustering (heatmap) and MDS plots support the clinical finding showing that GM173400 has a typical BIS episignature, and not a broad BAFopathy one (C), BIS probe set presenting case GM173400 (red), BIS cases (purple), BAFopathy cases (blue), controls (green). (D) BAFopathy probe set presenting case GM173400 (red), BIS cases (purple), CSS4_c.2656 (black), BAFopathy cases (blue), controls (green). (E) BIS probe set presenting case GM173400 (red), BIS cases (purple), CSS4_c.2656 (black), BAFopathy cases (blue), controls (green).

SMARCA2 missense variants cause two distinct syndromes depending on their location within the protein: variants in the catalytic SNF2 ATPase helicase domain cause NCBRS whereas variants outside of this domain cause BIS (Figure 4A). Sample GM173400 was part of the VUS cohort and had a *de novo SMARCA2*: c.2566A>G p.(M856V) missense substitution. The patient’s phenotype was compatible with BIS (Figures 4B; Table 3), but contrasted with the location of the variant within the SNF2 ATPase domain. The DNAm analysis matched the BIS episignature, which is clearly distinct from that of the BAFopathies (Figure 4C).

We also noted that the mDNA profile of GM173400 (SMARCA2 M856V) partially overlapped with a previously reported NDD with an underlying SMARCA4 M886V variant that was noted because its episignature was distinct from that of other SMARCA4 variants (Figures 4D and 4E). SMARCA4 M886V was considered a unique example of an episignature that is associated with a specific amino acid change.^10^

**Figure 5:**
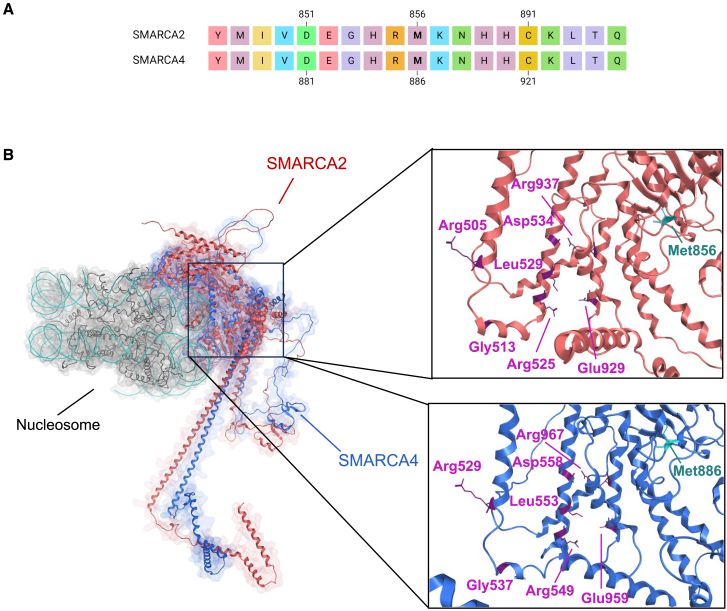
The similar episignature are exerted by homologous missense changes in *SMARCA2* and *SMARCA4* (A) Alignment of SMARCA2 and SMARCA4 paralogous proteins showing a 19 amino acid tract of complete amino acid identity centered on the conserved residue Met856. (B) Superimposed 3D structures of SMARCA2 (red) and SMARCA4 (blue). On the right, zoom in on the region containing SMARCA2 Met856 and SMARCA4 Met886 show they are isopositional. Several other structurally homologous amino acids of the two proteins are shown.

To explain this observation, alignment of the SMARCA2 and SMARCA4 protein sequences showed that SMARCA2 M856 and SMARCA4 M886 are positionally homologous amino acids (Figure 5A). This was confirmed by 3D protein homology modeling showing that these amino acids are indeed structural homologues, as is evident when the two protein structures are superposed (Figure 5B). This result supports the hypothesis that an identical M-to-V change in SMARCA2 at residue M856 or SMARCA4 at residue M886 exerts equivalent effects resulting in a shared episignatures.

### Validation of CNVs of uncertain significance in VCFS/DGS

We analyzed 11 CNVs of uncertain significance that do not span the typical 3-Mb or 1.5-Mb 22q11.21 deletions associated with VCFS/DGS (Figure S11A).^31^^,^^32^ Four CNVs consisted of variably sized deletions (approximately 304–772 kb) involving the 3′ terminal 22q11.22 VCFS/DGS region. As expected, none of these cases showed the VCFS/DGS profile nor any other known episignature profile (Figures S11B and S11C), confirming that the VCFS/DGS episignature is associated with haploinsufficiency of one or more genes at the 5′ end of the critical region. Sample S890 was a possible exception, with a methylation profile that was between VCFS/DGS cases and controls. We hypothesize that this case may have other genetic determinants that cause the DNAm profile to be closer to the VCFS/DGS episignature, also because this deletion is very similar to samples GM203534 and 140901 that have a DNAm profile as the control population. Indeed, S890 has two additional CNVs [GRCh37/hg19:9:127494563-127569992X3; GRCh37/hg19:2:135027917-136083735X3], which may contribute to the DNAm profile.

Finally, we analyzed seven cases with different 22q11.22 distal deletions. None of them showed the VCFS/DGS episignature (Figure S11), including two cases (GM151544 and GM191550) with distal low-copy-number repeat sequence (LCR)-DE deletions that included *TOP3B* (OMIM∗ 603582), which is associated with cognitive impairment and facial dysmorphisms.^33^ Three cases had an embedded deletion within this region, and two cases had distal LCR-EF deletions. In none of these cases did we detect the 22q11.2DS episignature.

## Discussion

The use of epigenetic signatures as biomarkers to validate VUS in clinical settings has received significant attention in recent years. Currently, there are more than 65 Mendelian disorders that are defined by specific episignatures.^10^ However, there are additional complexities emerging from the interpretation of episignature data. These complexities include (1) broad signals involving genes encoding different proteins that are part of multi-protein complexes; (2) sub-episignatures that are specific to gene protein domains; and (3) even sub-episignatures that are specific to single amino acid changes.^10^

VUS pose a challenge in rare genetic conditions, particularly in cases where the clinical presentation is ambiguous. Several cases in our cohort highlight the importance of using an epigenetic classifier to solve VUS. This method allows for the application of the PS3/BS3 functional evidence evaluation criteria within the clinical variant interpretation guidelines of the ACMG/AMP.^34^

Loss-of-function variants in *ARID1B* are associated with CSS1.^35^ These variants can include nonsense, frameshift, splice-site, and other deleterious structural changes.^36^^,^^37^ However, the role of missense variants in CSS1 is debated; it is suggested that such variants be interpreted with caution and are more likely to be considered harmless.^38^ Rare missense variants have been reported in the literature and considered pathogenic because they are *de novo*, but without functional evidence to support this assumption.^38^^,^^39^ In our study, we identified a *de novo ARID1B* missense variant (c.2480C>T p.(A827V)) that was confirmed as pathogenic through episignature analysis, confirming that missense variants in *ARID1B* can indeed cause CSS1. The availability of this rapid test, which can distinguish pathogenic from benign missense changes in *ARID1B*, is an important addition to the tools available for diagnosing CSS1, especially considering that defects in *ARID1B* are the main genetic cause of corpus callosum anomalies in patients with intellectual disability.^39^

Another example of the discriminating power of episignatures comes from patient NWM-116D with a potentially pathogenic splicing variant in *BRWD3*. Although the clinical presentation was consistent with BRWD3-associated intellectual disability, further evaluation was needed to determine if the variant was pathogenic. Splicing variants can be studied using different techniques, such as expression analysis or *in vitro* minigene splicing assays.^40^ In our case, in which patient-derived tissue was unavailable, episignature analysis not only represented a practical means for assessing the impact of the variant, but it also confirmed the pathogenicity of the variant and resolved the case.

Episignature analysis has not only been used to classify patients with VUS, but also to reclassify patients who were initially diagnosed incorrectly or to confirm a clinical suspicion when a predicted causative variant is not detected. In our case, episignature analysis supported the clinical diagnosis in two patients suspected of being CdLS (150163 and NWM-236D), but with no evidence of causative variants in the five genes so far identified as underlying this syndrome. It is known that pathogenic variants in *NIPBL*, *SMC1A*, *SMC3*, *RAD21*, and *HDAC8* explain about 65% of CdLS cases, suggesting that other genes (or variants in non-coding regions) are involved.^41^ Data from the literature indicate that deep-intronic and 5′ UTR variants in *NIPBL* can also cause CdLS.^42^^,^^43^^,^^44^^,^^45^^,^^46^ Therefore, we conducted genome sequencing on case 150163 and thoroughly analyzed the CdLS genes, including introns and non-translated regions, but we could not identify a possible pathogenic variant. This leaves open the possibility of a novel gene causing CdLS. If this hypothesis is true, the novel CdLS gene is likely to encode a protein in the same pathway as the known CdLS genes. Nonetheless, our findings suggest that episignature profiling can be used to support the diagnosis of CdLS even before conducting genetic screening in individuals with a clinical suspicion of CdLS.

In a case of MRXFH1 associated with *ATRX* (111092), episignature analysis also supported the clinical diagnosis despite the absence of any potentially causative single SNV in the gene. This case was further investigated, and ultimately a genomic deletion spanning *ATRX* exons 3–4 was identified, definitively confirming the presence of this disorder.

CNVs represent a significant proportion of the variants that cause NDDs. The changes occurring in DNAm profiles in patients with pathogenic CNVs have not yet been studied systematically, although there are reports of episignatures associated with pathogenic CNVs. In our study, episignature profiling confirmed that all the tested CNVs were indeed pathogenic. The 22q11.2 deletion syndrome is the most common microdeletion syndrome.^32^ It is characterized by high phenotypic variety and a variety of deletion types and sizes in the 22q11.2 region, which is due to several LCRs (LCR22). A 2.54-Mb deletion is the most common, accounting for approximately 90% of cases. There are also other deletions, such as a 1.5-Mb heterozygous deletion extending from LCR A-B (proximal deletion), a deletion extending from LCR A-C, and smaller atypical (nested) heterozygous deletions extending from LCR B-D or C-D, known as central deletions. Less frequently, distal deletions flanked by LCR D-E and LCR D-F have been reported, which did not show a specific methylation profile.

The most interesting data came from cases where the episignature profiles were different from what was expected. The analysis of patient GM184039, who had a likely pathogenic splicing variant in *EP300* (c.3671+5G>C), strongly indicated RSTS. However, the clustering of the data provided more support for RSTS1 (OMIM: 180849) rather than the expected RSTS2 (OMIM: 613684). This could indicate episignature limitations derived from the interference of the two epigenetic conditions (EP300: c.3671+5G>C; GNAS A/B:TSS-DMR), or that different variants in *EP300* may result in different DNAm profiles, as has been shown for other genes. For example, there is a specific signature called CSS_c.6200 that is associated with variants in the terminal region of *ARID1B* or *ARID1A*.^10^ Additionally, there are domain-specific DNAm episignatures in *ADNP*,^14^ a distinct DNAm signature in SRCAP associated with Floating-Harbor syndrome (FLHS) compared with non-FLHS SRCAP-related NDD,^47^ and finally a unique CSS4_c.2656 variant-specific episignature.^10^ Concerning these sub-episignatures, our data strongly support the hypothesis that they are associated with functional 3D domains. We found that the CSS_c.6200 episignature is also shared by variants SMARCB1:Gly11Arg and SMARCB1:Arg37His. Three-dimensional protein modeling of the BAF complex revealed that all the known amino acid changes associated with the CSS_c.6200 episignature in ARID1A/B and SMARCB1 are located within close proximity in the DNA-binding domain, specifically the SMARCB1 N-terminal helix and ARM domain of ARID1A (Figure 3E).^23^ This provides further evidence that these amino acid changes have a shared altered function, leading to similar phenotypes and methylation patterns.^28^

The presence of a *de novo SMARCA2* c.2566A>G p.(M856V) variant in the NCBRS-associated domain in patient GM173400, who has BIS and a consistent methylation profile, could be explained using a similar rationale. The SMARCA2:M856V and SMARCA4:M886V variants are structurally identical, and these two proteins are mutually exclusive in the complex. This not only expands the *SMARCA4* c.2656A>G sub-signature to include another variant, but also further supports that specific episignatures are associated with a 3D domain and its function. Furthermore, the recurrent SMARCA2 R855P change, which is located just one amino acid upstream of Met856, has been observed in patients with NCBRS and its associated BAF-methylation profile.

It is likely that these episignature-associated domains converge and contribute to a shared function, which ultimately influences the observed phenotypes and methylation patterns. This highlights the importance of considering the 3D organization of proteins and their interactions within complexes when studying the functional impact of amino acid changes and their association with specific signatures.

A final consideration is relative to the methylation profiles determined by variants on the X chromosome. In a female (NWM-024) with mild Borjeson-Forssman-Lehmann syndrome,^27^^,^^48^^,^^49^ associated with *de novo* PHF6 p.(C297F),^17^ we did not find the expected *PHF6* episignature. We suggest that this gene has sex-related episignature depending on whether female or male patients are analyzed. In fact, patients used to generate the episignature for *PHF6* were only males. Alternatively, a domain-specific episignature may exist since our patient’s change resides in the PHD2 domain where all reported missense variants in females are located (Figure S3). The role of skewed XCI in determining the epigenetic profile should also be considered as female cases with CdLS5 (OMIM: 300882) (*HDAC8* gene) with completely skewed X-inactivation did not show any change in their methylation profile.^9^

This interplay between an X-linked condition and episignatures could be also observed in another family where the *KDM5C* p.(D402N) change segregated in a mildly affected male, and two unaffected females. Notably, codon 402 has been reported to be changed to Tyr in other MRXSCJ patients and experimentally confirmed as deleterious.^50^ We have previously examined this family using XCI and linkage analysis^17^ and we showed that the mother tended to inactivate the mutant allele, while the affected sister had the wild type allele. Methylation analysis in the male proband 121116 computed an MVP score of 0.71, suggesting on a DNAm profile more similar to carrier females than affected males; his sister 121886 had an MVP of 0.54 with a DNAm profile similar to carrier females; and the carrier mother 121888 had an MVP score of 0.11, i.e., with a methylation profile like that of the control population, overall suggesting the variant is hypomorphic, and XCI is modulating the DNAm profile influencing protein levels. These findings are in line with the reported linear relationship seen between the dosage of the defective protein and the intensity of DNAm alterations in other syndromes, such as immunodeficiency-centromeric instability-facial anomalies syndrome types 2–4 (ICF2–4).^9^

Patient NWM-021D had an unusual finding with skewed XCI and DNAm pattern, which corresponds with two non X-linked genes, *ANKRD11* (KBG) (OMIM: 148050) and *SETD5* (MRD23) (OMIM: 615761). From a clinical perspective, the patient does not perfectly match with either of these conditions. However, the literature suggests that *ANKRD11* is a more likely candidate due to the involvement of its protein in XCI, specifically its interaction with HDAC3, a component of the XCI mechanism.^51^^,^^52^ It is also interesting to note that KBG is more common in males (male to female ratio 21:8) and initially it was proposed that *ANKRD11* had an X-linked inheritance.^53^^,^^54^ It would thus be of great interest to investigate how many autosomal genes play a role in XCI and how this may impact episignature interpretation.

### Conclusions

Using the EpiSign v.3 classifier we have highlighted the role of episignatures in solving VUS within a cohort of NDD cases. The integrated EpiSign/ES approach was helpful for re-evaluating already solved cases, for reclassifying variants of dubious clinical significance, and for detecting underlying genetic causes. Finally, we provide novel insights into sub-domain episignatures of the BAF complex, showing that they correlate with 3D functional domains. Despite current limitations of the size of its gene catalog, the Episign classifier is a powerful addition to the geneticist’s armamentarium, capable of obtaining returnable genetic results, especially in NDD patients.

## Data and code availability

The data supporting the findings of this study are available from the corresponding author. All variants have been deposited into ClinVar (SUB13925176); variants were validated with Variant Validator.

Some of the datasets used in this study are publicly available and may be obtained from the gene expression omnibus (GEO) using the following accession numbers: GEO: GSE116992, GSE66552, GSE74432, GSE97362, GSE116300, GSE95040, GSE104451, GSE125367, GSE55491, GSE108423, GSE116300, GSE89353, GSE52588, GSE42861, GSE85210, GSE87571, GSE87648, GSE99863, and GSE35069. These include DNAm data from patients with Kabuki syndrome, Sotos syndrome, CHARGE syndrome, immunodeficiency-centromeric ICF syndrome, Williams-Beuren syndrome, Chr7q11.23 duplication syndrome, BAFopathies, Down syndrome, a large cohort of unresolved subjects with developmental delays and congenital abnormalities, and several large cohorts of DNAm data from the general population. The rest of the data including the FA samples are not available due to the institutional or REB restrictions. EpiSign is a proprietary, trademarked analytical software owned by EpiSign Inc. Parts of it are based on the methods and publicly available software that are referenced in the Methods.

## Web resources

AlphaFold DB (https://alphafold.ebi.ac.uk) was employed for the retrieval of full-length predicted protein structures. Templates for the models of the human protein complexes were retrieved from the Protein DataBank (https://www.rcsb.org). Preliminary analysis of contact residues was performed with the webserver PDBSum Generate (https://www.ebi.ac.uk/thornton-srv/databases/pdbsum/Generate.html). Last access to the webservers and database: June 2nd, 2023. EpiSign v.3: https://episign.lhsc.on.ca/.

## Supplemental information

Document S1. Figures S1–S11, Tables S1–S3 and Supplemental Materials and methods

Document S2. Article plus supplemental information
