# Supplementary material for: DNA methylation analysis in patients with neurodevelopmental disorders improves variant interpretation and reveals complexity
Source: HGG Adv. 2024 May 15;5(3):100309. doi: 10.1016/j.xhgg.2024.100309 (PMC11216013; doi:10.1016/j.xhgg.2024.100309)
Supplement: Document S1. Figures S1–S11, Tables S1–S3 and Supplemental Materials and methods [file mmc1.pdf]

HGGA, Volume *5*

Supplemental information

DNA methylation analysis in patients with neurodevelopmental disorders improves variant interpretation and reveals complexity

Slavica Trajkova, Jennifer Kerkhof, Matteo Rossi Sebastiano, Lisa Pavinato, Enza Ferrero, Chiara Giovenino, Diana Carli, Eleonora Di Gregorio, Roberta Marinoni, Giorgia Mandrile, Flavia Palermo, Silvia Carestiato, Simona Cardaropoli, Verdiana Pullano, Antonina Rinninella, Elisa Giorgio, Tommaso Pippucci, Paola Dimartino, Jessica Rzasa, Kathleen Rooney, Haley McConkey, Aleksandar Petlichkovski, Barbara Pasini, Elena Sukarova-Angelovska, Christopher M. Campbell, Kay Metcalfe, Sarah Jenkinson, Siddharth Banka, Alessandro Mussa, Giovanni Battista Ferrero, Bekim Sadikovic, and Alfredo Brusco

SUPPLEMENTAL INFORMATION

- Supplemental figures S1-S11

- Supplemental tables 1-3

- Supplemental Materials and methods

- Supplemental References

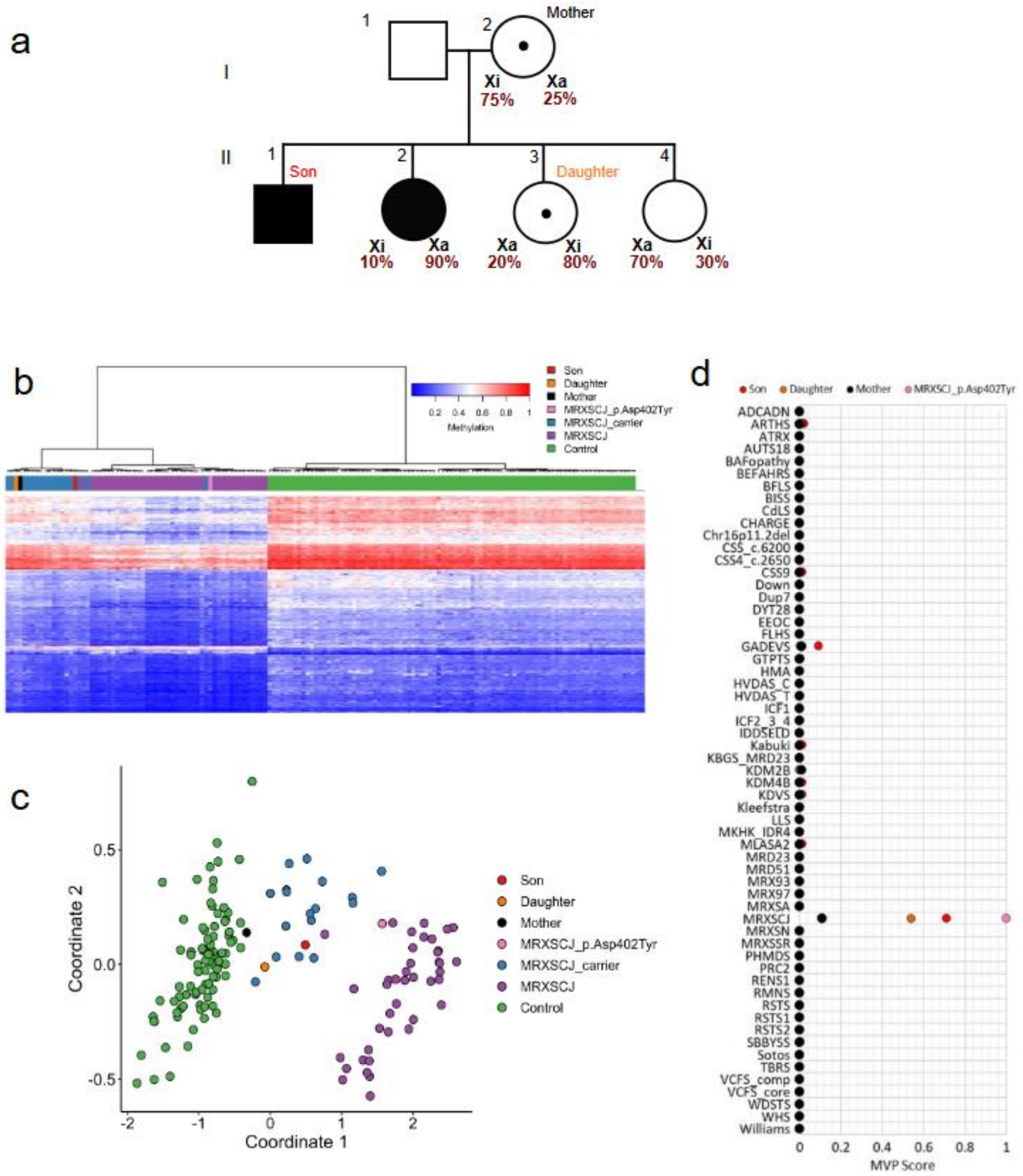

Supplemental figure S1. Family tree of *KDM5C* cases and EpiSign analysis

Panel a. Family tree and X-chromosome inactivation analysis (for further details please see[1]). **Panel b.** Euclidean hierarchical clustering (heatmap) of MRXSCJ-male cases (purple), MRXSCJ-female carriers (blue), green (controls); red-son (II-1); orange-daughter (II-3), black-mother (I-2) pink-male case MRXSCJ**:** p.(D402Y). **Panel c**. Multidimensional scaling (MDS) plot presents the differentiation of MRXSCJ-male cases (purple), MRXSCJ-female carriers (blue), green (controls); red-son (II-1); orange-daughter (II-3), black-mother (I-2); pink-male case MRXSCJ**:** p.(D402Y). **Panel d**-MVP score plots orange-daughter (II-3), black-mother (I-2), red-son (II-1); pink-male case MRXSCJ**:**p.(D402Y).

Alignments:

73.9% identity in 1672 residues overlap; Score: 5747.0; Gap frequency: 6.5%

```
P51531|SMC     1 MSTPTDP-GAMPHPGPSPGPGPSPGPILGPSPGPGPSPGSVHSMMGPSPGPPSVSHPMPT
P51532|SMC     1 MSTPDPPLGGTPRPGPSPGPGPSPGAMLGPSPGP--SPGSAHSMMGPSPGPPSAGHPIPT
                 ****  * *  * ************  *******  **** ************  ** **

P51531|SMC    60 MGSTDFPQEGMHQMHKPIDGIHDKGIVEDIHCGSMKGTGMRPP-HPGMGPPQSPMDQHSQ
P51532|SMC    59 QGPGGYPQDNMHQMHKPMESMHEKGMSDDPRYNQMKGMGMRSGGHAGMGPPPSPMDQHSQ
                  *    **  *******    * **   *     *** ***   * ***** ********

P51531|SMC   119 GYMSPHPSPLGAPEHVSSPMSGGGPTP-PQMPPSQPGALIPG-DPQAMSQPNRGPSPFSP
P51532|SMC   119 GY----PSPLGGSEHASSPVPASGPSSGPQMSSGPGGAPLDGADPQALGQQNRGPTPFNQ
                 **    *****  ** ***    **   ***     **   * ****  * **** **

P51531|SMC   177 VQLHQLRAQILAYKMLARGQPLPETLQLAVQGKRTLPGLQQQQQQQQQQQQQ--------
P51532|SMC   175 NQLHQLRAQIMAYKMLARGQPLPDHLQMAVQGKRPMPGMQQQMPTLPPPSVSATGPGPGP
                  ********* ************  ** ******  ** ***

P51531|SMC   229 -------------QQQQQQQQQQPQQQPPQPQT----QQQQQPALVNYNRPSGPGPELSG
P51532|SMC   235 GPGPGPGPGPAPPNYSRPHGMGGPNMPPPGPSGVPPGMPGQPPGGPPKPWPEGPMANAAA
                                        *   ** *         * *       * **

P51531|SMC   272 P-STPQKLPVPAPGGRPSPAPPAAAQPPAAAVPGPSVPQPAPGQPSPVLQLQQKQSRISP
P51532|SMC   295 PTSTPQKLIPPQPTGRPSPAPPAVPPAASPVMPPQTQSPGQPAQPAPMVPLHQKQSRITP
                 * ******  * * *********         *        * ** *   * ****** *

P51531|SMC   331 IQKPQGLDPVEILQEREYRLQARIAHRIQELENLPGSLPPDLRTKATVELKALRLLNFQR
P51532|SMC   355 IQKPRGLDPVEILQEREYRLQARIAHRIQELENLPGSLAGDLRTKATIELKALRLLNFQR
                 **** *********************************  ******* ************

P51531|SMC   391 QLRQEVVACMRRDTTLETALNSKAYKRSKRQTLREARMTEKLEKQQKIEQERKRRQKHQE
P51532|SMC   415 QLRQEVVVCMRRDTALETALNAKAYKRSKRQSLREARITEKLEKQQKIEQERKRRQKHQE
                 ******* ****** ****** ********* ***** **********************

P51531|SMC   451 YLNSILQHAKDFKEYHRSVAGKIQKLSKAVATWHANTEREQKKETERIEKERMRRLMAED
P51532|SMC   475 YLNSILQHAKDFKEYHRSVTGKIQKLTKAVATYHANTEREQKKENERIEKERMRRLMAED
                 ******************* ****** ***** *********** ***************

P51531|SMC   511 EEGYRKLIDQKKDRRLAYLLQQTDEYVANLTNLVWEHKQAQAAKEKKKRRRRKKKAEENA
P51532|SMC   535 EEGYRKLIDQKKDKRLAYLLQQTDEYVANLTELVRQHKAAQVAKEKKKKK--KKKKAENA
                 ************* ***************** **  ** ** ******    ***  ***

P51531|SMC   571 EGGESALGPDGEPIDESSQMSDLPVKVTHTETGKVLFGPEAPKASQLDAWLEMNPGYEVA
P51532|SMC   593 EGQTPAIGPDGEPLDETSQMSDLPVKVIHVESGKILTGTDAPKAGQLEAWLEMNPGYEVA
                 **   * ****** ** ********** * * ** * *  **** ** ************

P51531|SMC   631 PRSDSEESDSDYEEEDEEEESSRQET-------EEKILLDPNSEEVSEKDAKQIIETAKQ
P51532|SMC   653 PRSDSEESGSEEEEEEEEEEQPQAAQPPTLPVEEKKKIPDPDSDDVSEVDARHIIENAKQ
                 ******** *  *** ****             * *   ** *  *** **  *** ***

P51531|SMC   684 DVDDEYSM-QYSARGSQSYYTVAHAISERVEKQSALLINGTLKHYQLQGLEWMVSLYNNN
P51532|SMC   713 DVDDEYGVSQALARGLQSYYAVAHAVTERVDKQSALMVNGVLKQYQIKGLEWLVSLYNNN
                 ******   *  *** **** ****  *** *****  ** ** **  **** *******

P51531|SMC   743 LNGILADEMGLGKTIQTIALITYLMEHKRLNGPYLIIVPLSTLSNWTYEFDKWAPSVVKI
P51532|SMC   773 LNGILADEMGLGKTIQTIALITYLMEHKRINGPFLIIVPLSTLSNWAYEFDKWAPSVVKV
                 ***************************** *** ************ ************

P51531|SMC   803 SYKGTPAMRRSLVPQLRSGKFNVLLTTYEYIIKDKHILAKIRWKYMIVDEGHRMKNHHCK
P51532|SMC   833 SYKGSPAARRAFVPQLRSGKFNVLLTTYEYIIKDKHILAKIRWKYMIVDEGHRMKNHHCK
                 **** ** **  ************************************************

P51531|SMC   863 LTQVLNTHYVAPRRILLTGTPLQNKLPELWALLNFLLPTIFKSCSTFEQWFNAPFAMTGE
P51532|SMC   893 LTQVLNTHYVAPRRLLLTGTPLQNKLPELWALLNFLLPTIFKSCSTFEQWFNAPFAMTGE
                 ************** *********************************************

P51531|SMC   923 RVDLNEEETILIIRRLHKVLRPFLLRRLKKEVESQLPEKVEYVIKCDMSALQKILYRHMQ
P51532|SMC   953 KVDLNEEETILIIRRLHKVLRPFLLRRLKKEVEAQLPEKVEYVIKCDMSALQRVLYRHMQ
```

```
                  ******************************** ******************  ******

P51531|SMC   983 AKGILLTDGSEKDKKGKGGAKTLMNTIMQLRKICNHPYMFQHIEESFAEHLGYSNGVING
P51532|SMC  1013 AKGVLLTDGSEKDKKGKGGTKTLMNTIMQLRKICNHPYMFQHIEESFSEHLGFTGGIVQG
                 *** *************** *************************** ****   *   *

P51531|SMC  1043 AELYRASGKFELLDRILPKLRATNHRVLLFCQMTSLMTIMEDYFAFRNFLYLRLDGTTKS
P51532|SMC  1073 LDLYRASGKFELLDRILPKLRATNHKVLLFCQMTSLMTIMEDYFAYRGFKYLRLDGTTKA
                   *********************** ******************* * * *********

P51531|SMC  1103 EDRAALLKKFNEPGSQYFIFLLSTRAGGLGLNLQAADTVVIFDSDWNPHQDLQAQDRAHR
P51532|SMC  1133 EDRGMLLKTFNEPGSEYFIFLLSTRAGGLGLNLQSADTVIIFDSDWNPHQDLQAQDRAHR
                 ***  *** ****** ****************** **** ********************

P51531|SMC  1163 IGQQNEVRVLRLCTVNSVEEKILAAAKYKLNVDQKVIQAGMFDQKSSSHERRAFLQAILE
P51532|SMC  1193 IGQQNEVRVLRLCTVNSVEEKILAAAKYKLNVDQKVIQAGMFDQKSSSHERRAFLQAILE
                 ************************************************************

P51531|SMC  1223 HEEENE---------------------------------EEDEVPDDETLNQMIARREEE
P51532|SMC  1253 HEEQDESRHCSTGSGSASFAHTAPPPAGVNPDLEEPPLKEEDEVPDDETVNQMIARHEEE
                 ***  *                                 ********** ****** ***

P51531|SMC  1250 FDLFMRMDMDRRREDARNPKRKPRLMEEDELPSWIIKDDAEVERLTCEEEEEKIFGRGSR
P51532|SMC  1313 FDLFMRMDLDRRREEARNPKRKPRLMEEDELPSWIIKDDAEVERLTCEEEEEKMFGRGSR
                 ******** ***** ************************************** ******

P51531|SMC  1310 QRRDVDYSDALTEKQWLRAIEDGNLEEMEEEVRLKKRKRRRNVDKDPA------------
P51532|SMC  1373 HRKEVDYSDSLTEKQWLKAIEEGTLEEIEEEVRQKKSSRKRKRDSDAGSSTPTTSTRSRD
                  *  ***** ******* *** * *** ***** **  * *  * *

P51531|SMC  1358 KEDVEKAKKRRGRPPAEKLSPNPPKLTKQMNAIIDTVINYKDRCNVEKVPSNSQLEIEGN
P51532|SMC  1433 KDDESKKQKKRGRPPAEKLSPNPPNLTKKMKKIVDAVIKYKD-----------------S
                 * *  *  * ************** *** *  * * ** ***

P51531|SMC  1418 SSGRQLSEVFIQLPSRKELPEYYELIRKPVDFKKIKERIRNHKYRSLGDLEKDVMLLCHN
P51532|SMC  1476 SSGRQLSEVFIQLPSRKELPEYYELIRKPVDFKKIKERIRNHKYRSLNDLEKDVMLLCQN
                 *********************************************** ********** *

P51531|SMC  1478 AQTFNLEGSQIYEDSIVLQSVFKSARQKIAKEEESEDESNEEEEEEDEEESESEAKSVKV
P51532|SMC  1536 AQTFNLEGSLIYEDSIVLQSVFTSVRQKIEKEDDSEGEESEEEEEGEEEGSESESRSVKV
                 ********* ************ * **** **  ** *  *****  ** ****  ****

P51531|SMC  1538 KIKLNKKDDKGRDKGKGKKRPNRG-KAKPVVSDFDSDEEQDEREQSEGSGTD
P51532|SMC  1596 KIKLGRKEKAQDRLKGGRRRPSRGSRAKPVVSDDDSEEEQEEDRSGSGSEED
                 ****  *         *  ** **  ******* ** *** *     **  *
```

Supplemental figure S2: Multiple sequence alignment (MSA) between human SMARCA2 and SMARCA4 proteins.

Sequence alignment between human SMARCA2 (P51531) and SMARCA4 (P51532) proteins by SIM - Alignment Tool for Protein Sequences (https://web.expasy.org/sim/) using preset parameters. The alignment shows a 73.9% identity in 1672 residues overlap.

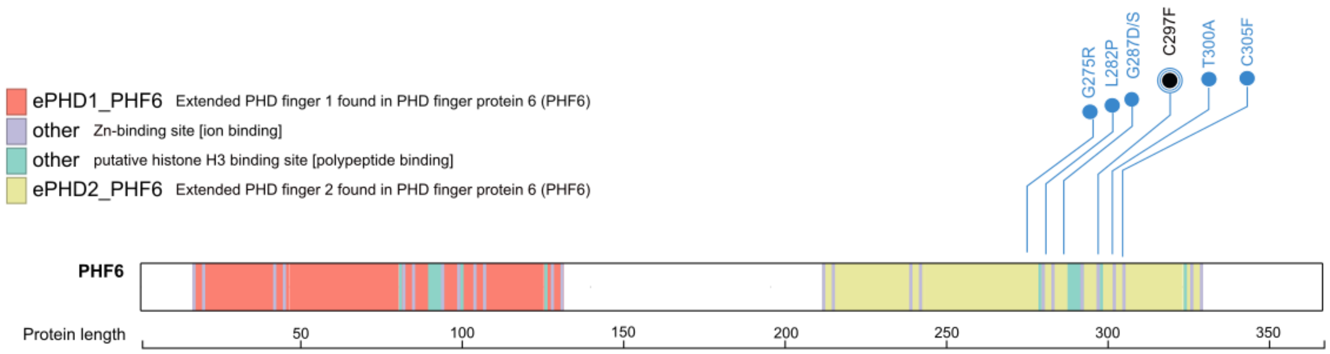

Supplemental figure S3: Reported missense variants in *PHF6* affected females.
Schematic drawing of literature reported missense variants in *PHF6* gene (NM_001015877)[13], using PeCan, St. Jude Cloud (https://pecan.stjude.cloud) software.

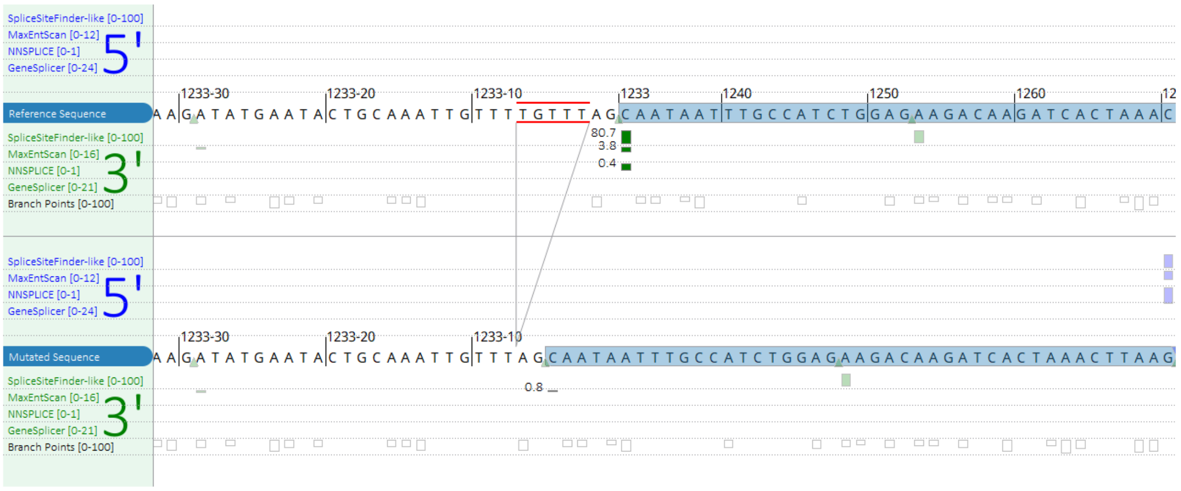

Supplemental figure S4: Impact on splicing of the NM_153252.5: c.1233-7_1233-3 variant in *BRWD3*
The impact of the NM_153252.5: c.1233-7_1233-3 variant in *BRWD3* was computed using AlamutVisualPlus software (ver1.7.1). The change is likely to affect the acceptor splice site of exon 14/41 as predicted by at least three softwares (MaxEnt: -79.8%; NNSPLICE: -99.4%; SSF: -19.2%; overall -66.1%). The consequence of this change on the mRNA is however to be tested experimentally on cDNA from the patient.

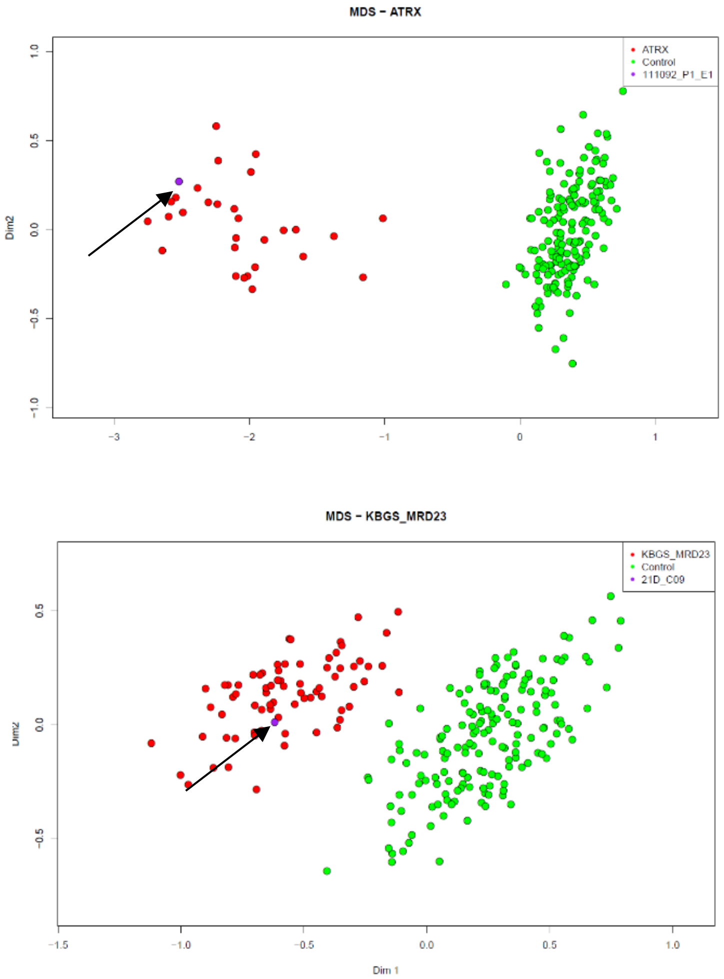

Supplemental figure S5: MDS plots for ATRX and KBG & MRD23 episignature profiling

Multidimensional scaling (MDS) plots: upper panel- ATRX gene (MIM# 301040); green: controls, red : cases, purple: case 111092, lower panel- ANKRD11 (KBG MIM#148050) & SETD5 (MRD23 MIM #615761); green :controls, red :cases, purple: case NWM-021D.

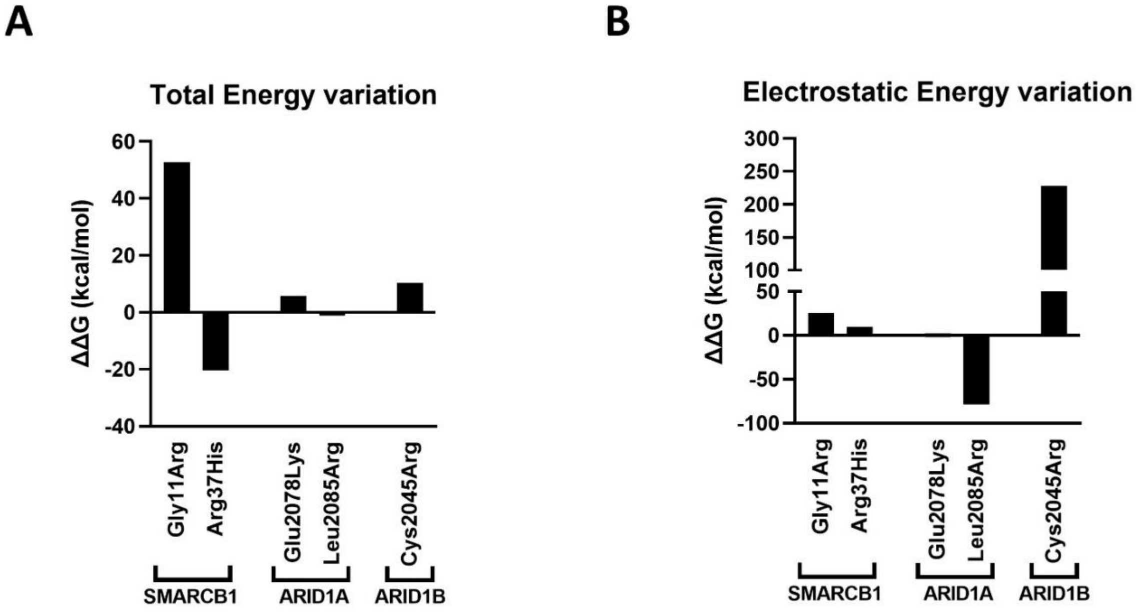

Supplemental figure S6 Energies calculated on the PDB complex (based on PDB id 6LTH)

Energy variation upon mutation and minimization (mutant-WT) estimated with the forcefield AMBER 12: EHT. Brackets below indicate which protein is the mutant product in the complex. A) is the sum of all energy terms, B) considers just the electrostatic term.

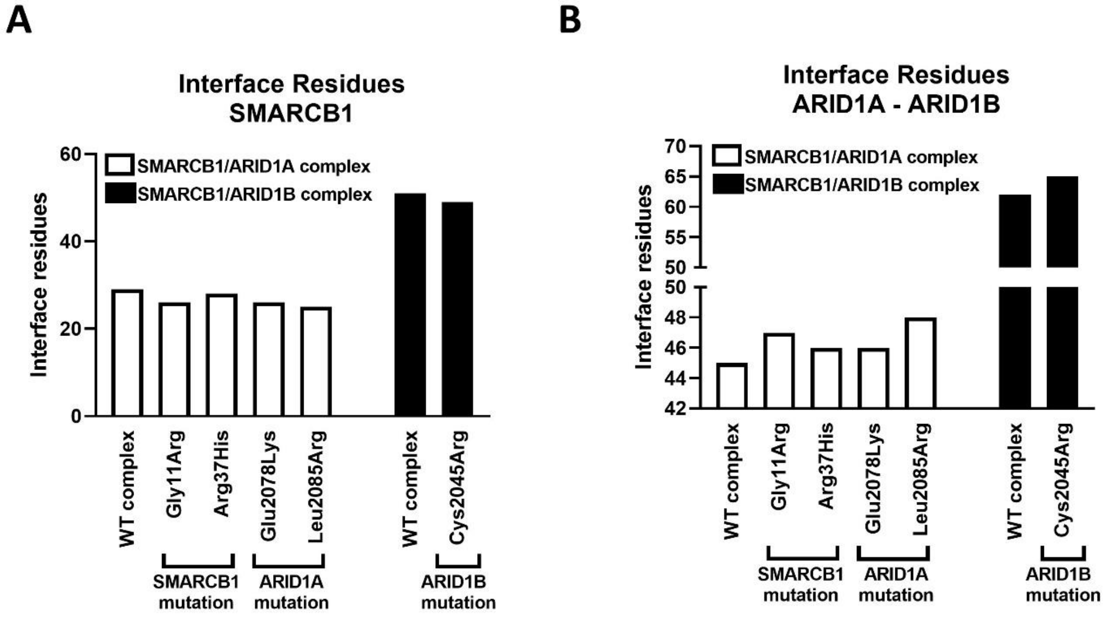

Supplemental figure S7: Residues at the complex interface (based on PDB id 6LTH)

Number of residues present at the interface between the proteins (SMARCB1/ARID1A, and SMARCB1/ARID1B complex). A) SMARCB1 residues, B) ARID1A/ARID1B residues.

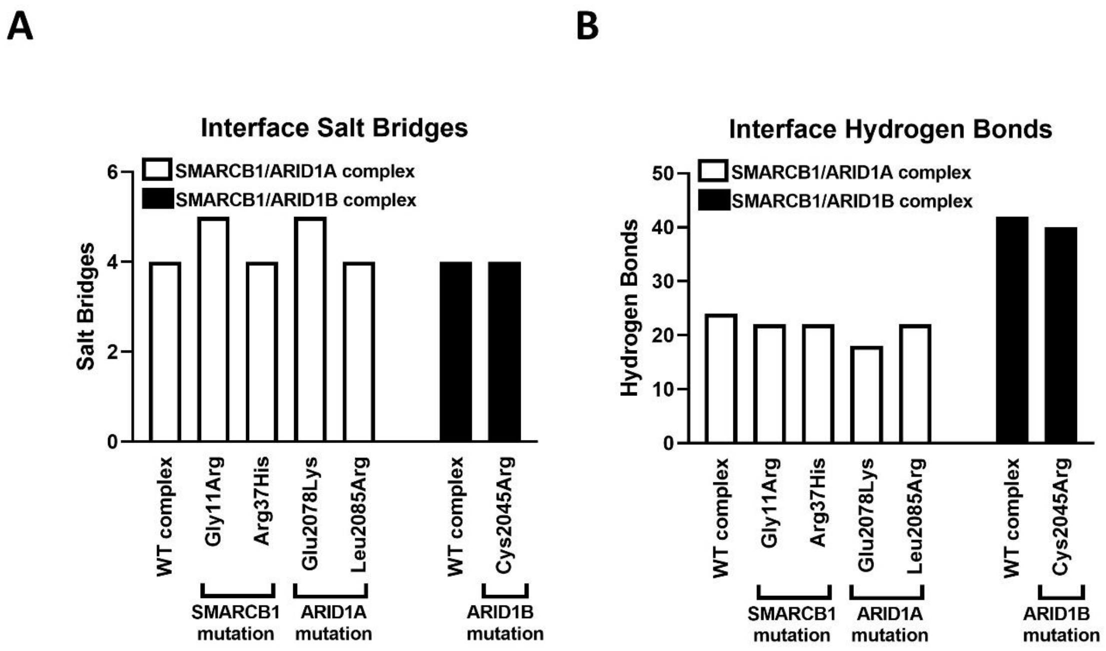

Supplemental figure S8: Interactions at the complex interface (based on PDB id 6LTH)
Number of Salt Bridges (A), and Hydrogen Bonds (B) at the interface between the proteins (SMARCB1/ARID1A, and SMARCB1/ARID1B complex).

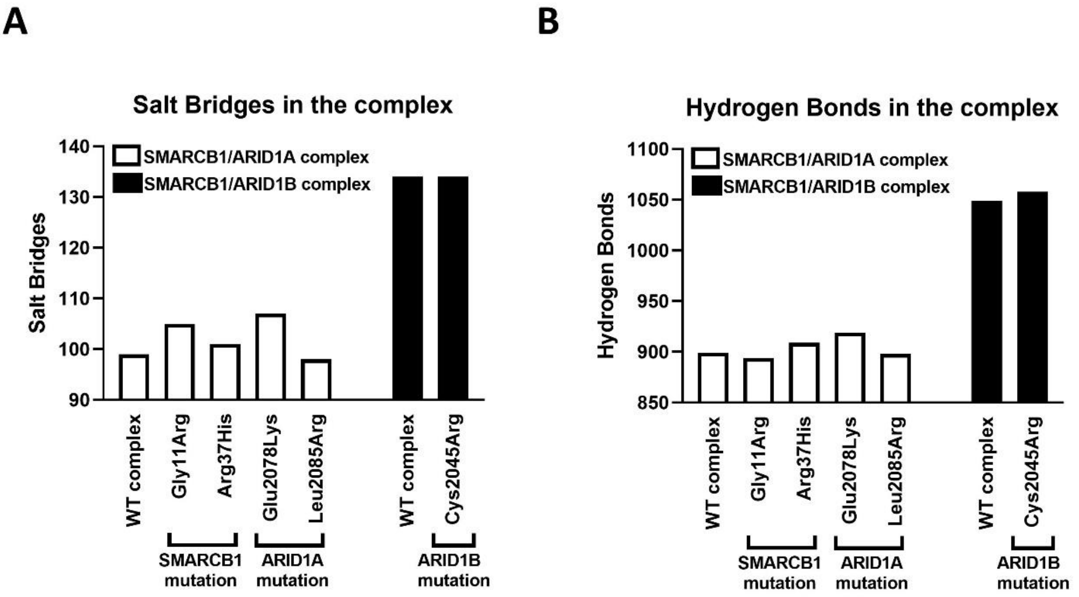

Supplemental figure S9: total interactions in the complex (based on PDB id 6LTH)
Total number of Salt Bridges (A), and Hydrogen Bonds (B) in the whole complex.

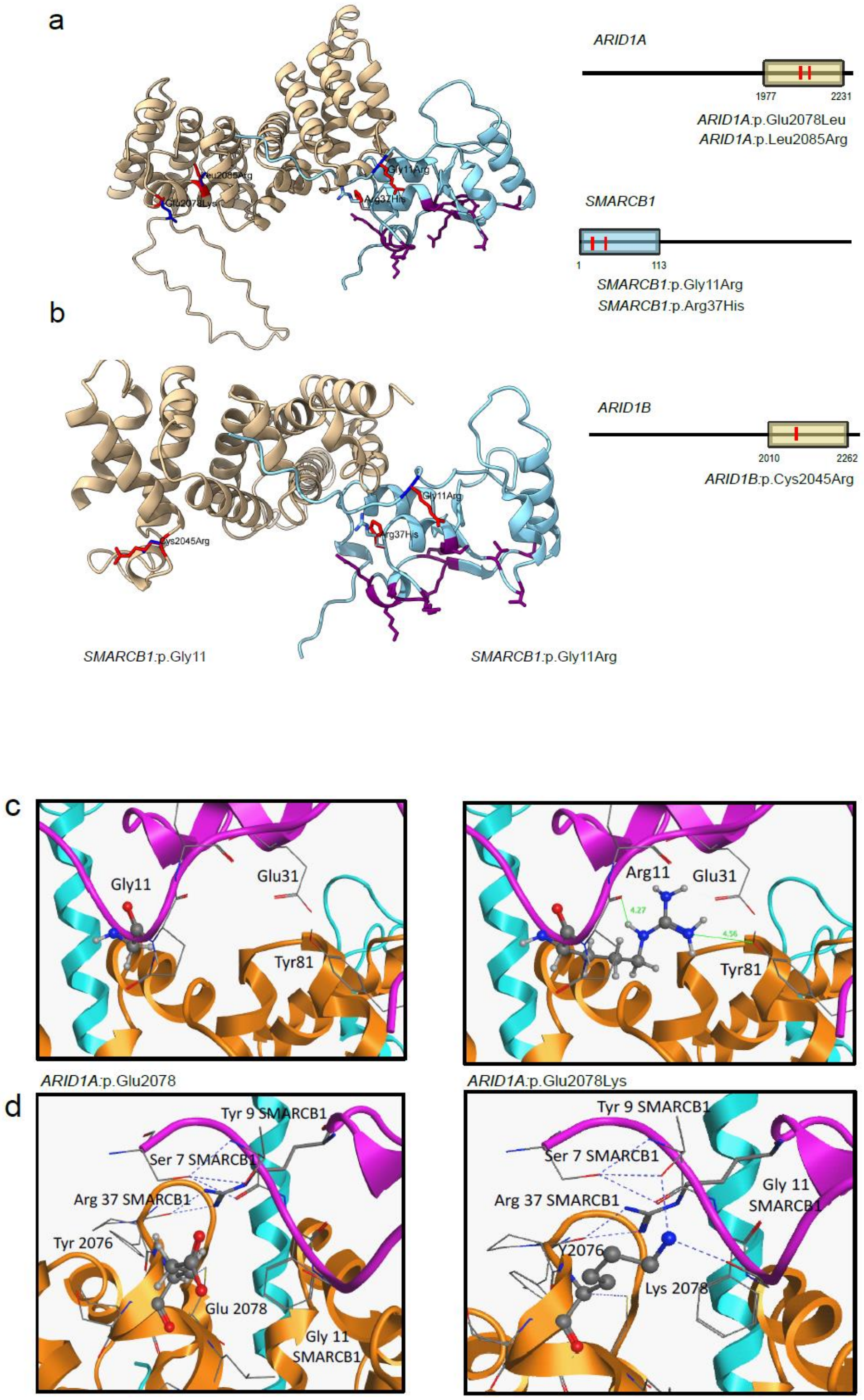

Supplemental figure S10. Comparison of ARID1A/ARID1B paralogues and SMARCB1 interacting amino acids.

Panel a: ARID1A-yellow-DUF3518 domain (a.a 1977-2231) SMARCB1-blue-DNA -binding domain (a.a 1-113) red:variant blue:wt purple:codons from DNA -binding domain of SMARCB1 that are in contact with DNA. **Panel b**: ARID1B-yellow-BAF250_C domain(a.a 2010-2262) red:variant blue:wt purple:codons from DNA -binding domain of SMARCB1 that are in contact with DNA;[14] (ARID1A- AlphaFold model:AF-O14497-F1, SMARCB1-AlphaFold model:F-Q12824-F1; ARID1B- AlphaFold model:AF-Q8NFD5-F1; modeled with UCSF ChimeraX version: 1.4 (www.cgl.ucsf.edu/chimerax) using the rotamers-tools function. **C)** Representative caption of the comparison between the sidechains of Gly11 SMARCB1 (WT), and Arg11

(Mutant) revealing the mutant residue involved in newly formed interactions. D) Caption of mutant p.(D2078K) SMARCB1 showing that the side chain of the mutant residue is inserted in an interaction (HB) network.

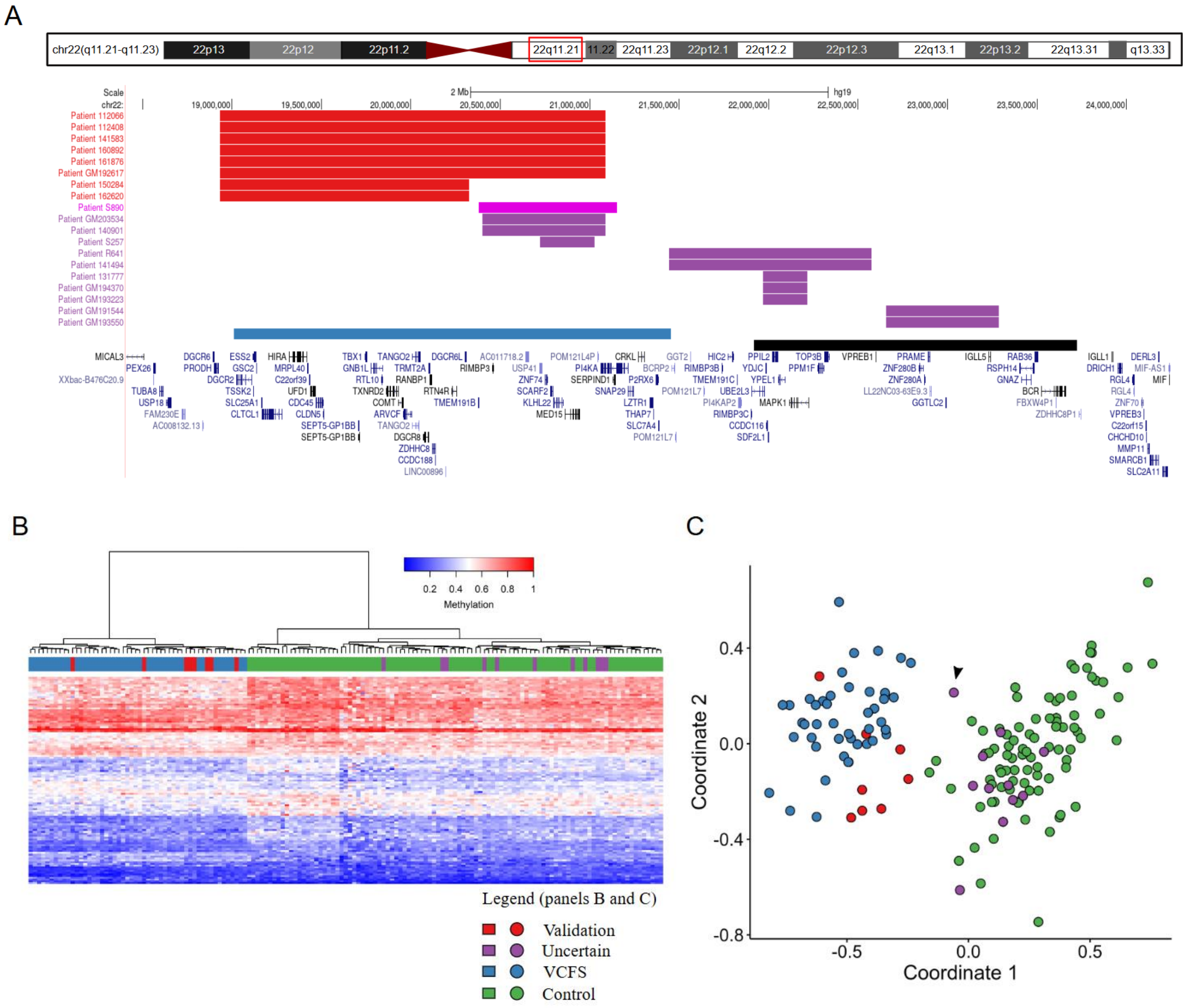

Supplemental figure S11. Epsignature analysis of CNVs in the 22q11.2 region.

A. Scheme of the 21 CNVs at 22q11.2 region reported in Table 1 (Validation cohort).

B, C. heatmap and MDS plot show that only the typical 22q11.2DS shows the associated Episignature profiles. Case S890 is clustering nearby 22q11.2DS cases, for unknown reasons (black arrow).

Supplemental Table 1. List of the cases analysed, ACMG/AMP variant classification and HPO terms

Sample ID	Sex	Phenotyp	Gene/ region involved	Ref Seq	Variant	ACMG/ AMP-criteria	classification/ score	HPO
**Validation cohort: Single Nucleotide Variants (SNVs) (34 cases)**								
**NWM-030D**	F	Helsmoortel-van der Aa syndrome	*ADNP*	NM_001282531.3	c.539_542del:p.(Val180fs)	PVS1; PM2;PP5	P	HP:0001252-Muscular hypotonia;HP:0001249-Intellectual disability
**GM223306**	F	Helsmoortel-van der Aa syndrome	*ADNP*	NM_001282531.3	c.2454C>G:p.(Tyr818Ter)	PVS1; PM2;PP5	P	HP:0001249-Intellectual disability; HP:0012758-Neurodevelopmental delay
**121623**	M	KBG syndrome	*ANKRD11*	NM_013275.6	c.439C>T:p.(Gln147*)	PVS1; PM2;PP5	P	HP:0001510-Growth delay, HP:0001156-Brachydactyly, HP:0000824-Decreased response to growth hormone stimulation test , HP:0011342-Mild global developmental delay, HP:0001629-Ventricular septal defect , HP:0000271-Abnormality of the face
**BA2012002**	F	KBG syndrome	*ANKRD11*	NM_013275.6	c.211_226+1del	PVS1; PM2;PP5	P	HP:0001249-Intellectual disability;HP:0011342-Mild global developmental delay,
**NWM-218D**	M	KBG syndrome	*ANKRD11*	NM_013275.6	c.1903_1907del:p.Lys635fs	PS4;PVS1; PM2;PP5	P	HP:0001249-Intellectual disability; HP:0001250-Seizures;HP:0001344-Absent speech;HP:0001290-Generalized hypotonia
**NMW-035D**	M	Coffin-Siris syndrome 2	*ARID1A*	NM_006015.6	c.6232G>A:p.(Glu2078Lys)	PS2;PM2;PP2;PP3	LP	HP:0001249; HP:0001655; HP:0001642;HP:0007376;HP:0002804;HP:00010311; HP:00028;HP:0001845;HP:00023;HP:0001290;HP:000767;HP:00030215;HP:000954;HP:000396;HP:000347;HP:000280; HP:000316;HP:000286; HP:00012810; HP:0002714;HP:000470;HP:000369;HP:00012385;HP:000474;HP:000582;HP:0006191;
**160759**	F	Coffin-Siris syndrome 1	*ARID1B*	NM_001374828.1	c.5825G>A:p.(Trp1942*)	PVS1; PS2; PM2	LP	HP:0001249-Intellectual disability;
**142220**	M	CHARGE syndrome	*CHD7*	NM_017780	c.3082A>G:p.(Ile1028Val)	PM1;PM2; PP2;PP3;PP5	LP	HP:0001249-Intellectual disability; HP:0008501-Median cleft lip and palate
**FS0208013**	M	CHARGE syndrome	*CHD7*	NM_017780	c.6194G>A:p.(Arg2065His)	PM1;PM2; PP2;PP3;PP5	LP	HP:0001249-Intellectual disability;
**GM110562**	M	Autism, susceptibility to	*CHD8*	NM_001170629.2	c.2025-1G>C	PVS1; PS2; PM2:PP5	LP	HP:0001249-Intellectual disability; HP:0001548-Overgrowth; HP:0000316-Hypertelorism; HP:0005280-Depressed nasal bridge; HP:0000286-Epicanthus; HP:0001263-Global developmental delay
**110212**	M	Rubinstein-Taybi syndrome 1	*CREBBP*	NM_004380.3	c.3779+1G>A	PVS1; PS2; PM2;PP5	P	HP:0001680-Coarctation of aorta; HP:0001647-Bicuspid aortic valve ; HP:0001633-Abnormal mitral valve morphology; HP:0001507-Growth abnormality;

**141444**	M	Kleefstra syndrome 1	*EHMT1*	NM_024757.5	c.3331T>A:p.(Cys1111Ser)	PS1;PS2;PM2;PP3	P	HP:0000729-Autistic behavior , HP:0006335-Persistence of primary teeth , HP:0000023-Inguinal hernia, HP:0000646-Amblyopia , HP:0001763- Pes planus, HP:0001263-Global developmental delay , HP:0000750, Stereotypy HP:0000733-Delayed speech and language development, HP:0001388-Joint laxity , HP:0000767-Pectus excavatum , HP:0007018-Attention deficit hyperactivity disorder, HP:0007057-Poor hand-eye coordination, HP:0000272-Malar flattening , HP:0000676-Abnormality of the incisor
**131361**	M	Kleefstra syndrome 1	*EHMT1*	NM_024757.5	c.3000del:p.(Asp1001fs)	PVS1; PS2; PM2;PP5	P	HP:0001643- Patent ductus arteriosus, HP:0001249-Intellectual disability; HP:0002870-Obstructive sleep apnea
**GM181933**	M	Kleefstra syndrome	*EHMT1*	NM_024757.5	c.508del:p.(Gln170fs)	PVS1; PS2; PM2;PP5	P	HP:0001263-Global developmental delay;HP:0001256-Intellectual disability,
**GM184039**	F	Rubinstein-Taybi syndrome 2	*EP300*	NM_001429.4	c.3671+5G>C	PS2; PS3;PM2;PM4;PP3	LP	HP:0001511-Intrauterine growth retardation; HP:0001561-Polyhydramnios , HP:0001518-Small for gestational age, HP:0011451-Primary microcephaly, HP:0001669-Transposition of the great arteries, , HP:0000365-Hearing impairment , HP:0001510-Growth delay, HP:0001263-Global developmental delay, HP:0000664-Synophrys , HP:0002553-Highly arched eyebrow, HP:0000470-Short neck, HP:0010711-1-2 toe syndactyly , HP:0025419-Pulmonary pneumatocele, HP:0005403-T lymphocytopenia
**NWM-019D**	M	Weaver syndrome	*EZH2*	NM_004456.5	c.2015T>G:p.(Phe672Cys)	PS2;PM1;PM2;PP2;PP3	LP	HP:0001249;HP:0008935;HP:0002721;HP:0001537;HP:00028;HP:0003037;HP:0005616;HP:0001655;HP:0004684;HP:000100806;HP:0004324;HP:000280;HP:000311;HP:0008070;HP:000256;HP:00011220;HP:0005469;HP:0001090;HP:000316;HP:000369;HP:0005280;HP:000343;HP:000218;HP:000277;HP:000470;HP:0001812;HP:00012385;HP:00030084;HP:0009381;HP:00010300;
**NWM-088D**	F	Rahman syndrome	*HIST1H1E*	NM_005321.3	c.458_460del:p.(Lys152fs)	PVS1; PM2;PP3	P	HP:0001263; HP:000717; HP:0002691; HP:00040194; HP:000280; HP:000337;HP:000490;HP:0007874;HP:000316;HP:000431; HP:000322; HP:0009765;HP:000455;HP:000303;HP:00040170;HP:0001182; HP:0007565;HP:000670;HP:000958;HP:000207;HP:0008070;
**GM201880**	F	Mental retardation, autosomal dominant 32	*KAT6A*	NM_006766.5	c.2927del:p.(Gly976Valfs)	PVS1;PS2; PM2	P	HP:0001263-Global developmental delay;HP:0001256-Intellectual disability,

**121116**	M	Intellectual developmental disorder, XL syndromic, Claes-Jensen type	*KDM5C*	NM_004187.5	c.1204G>A:p.(Asp402Asn)	PM2;PM5;PP2;PP5	LP	HP:0001249-Intellectual disability, HP:0000750-Delayed speech and language development;
**121886**	F	Intellectual developmental disorder, XL syndromic, Claes-Jensen type	*KDM5C*	NM_004187.5	c.1204G>A:p.(Asp402Asn)	PM2;PM5;PP2;PP5	LP	HP:0011342-Mild global developmental delay
**121888**	F	Intellectual developmental disorder, XL syndromic, Claes-Jensen type	*KDM5C*	NM_004187.5	c.1204G>A.(Asp402Asn)	PM2;PM5;PP2;PP5	LP	not affected
**NWM-192D**	F	WDSTS	*KMT2A*	NM_001197104.2	c.4777del:p.(Arg1593fs)	PVS1; PS2; PM2;PP5	P	HP:0001249-Intellectual disability, HP:0001518-Small for gestational age;HP:0000824-Growth hormone deficiency;HP:0000826-Precocious puberty;
**GM194228**	M	Kabuki syndrome 1	*KMT2D*	NM_003482.3	c.4395dup:p.(Lys1466fs)	PVS1, PM2, PP5	P	HP:0001249-Intellectual disability,
**NWM-031D**	F	Kabuki	*KMT2D*	NM_003482.3	c.13795_13802del:p.(Ala4599fs)	PVS1; PS2; PM2;PP3	P	HP:0001249; HP:0001319;HP:000343; HP:000337;HP:000316;HP:00012810;HP:000637;HP:0002553;HP:00011229;HP:000358;HP:0001212;HP:00010314
**NWM-024D**	F	Börjeson-Forssman-Lehmann syndrome	*PHF6*	NM_001015877.2	c.890G>T:p.(Cys297Phe)	PS2;PM1;PM2;PP2;PP3	LP	HP:0001263; HP:000717; HP:000175; HP:0001537;HP:0001290;HP:0001643;HP:0001156;HP:0004691;HP:000280;HP:000486;HP:000574;HP:000316;HP:000506;HP:000582;HP:000343;278;HP:000369;HP:000470;HP:000664;HP:00011229
**NWM-163D1**	M	Renpenning syndrome	*PQBP1*	NM_001032383.2	c.457_459del:p.(Arg153fs)	PVS1;PM2;PP3	P	HP:0001249-Intellectual disability,HP:0002194-Delayed gross motor development
**NWM-163D2**	M	Renpenning syndrome	*PQBP1*	NM_001032383.2	c.457_459del:p.(Arg153fs)	PVS1;PM2;PP3	P	HP:0001249-Intellectual disability,HP:0002194-Delayed gross motor development
**GM182051**	M	Renpenning syndrome	*PQBP1*	NM_001032383.2	c.233C>A:p.(Pro78Gln)	PM1; PM2;PM5; PP2; PP3;	LP	HP:0001250; HP:0010864; HP:0002415; HP:0001510; HP:0000118
**GM173348**	F	SETD1B-related syndrome	*SETD1B*	NM_001353345.2	c.598del:p.(Gln200fs)	PVS1; PS1; PS2; PM2; PP3	P	HP:0002342-Intellectual disability, moderate, HP:0012420-Meconium stained amniotic fluid, HP:0000750-Delayed speech and language development, HP:0001081-Cholelithiasis
**GM223349**	M	Intellectual developmental disorder,	*SETD5*	NM_001080517.3	c.868_872del:p.(Arg290fs)	PVS1;PS2;PM2	P	HP:0001249-Intellectual disability; HP:0001999-Abnormal facial shape, HP:0000047-Hypospadias, HP:0000028-Cryptorchidism

		autosomal dominant 23						
**GM223350**	F	Intellectual developmental disorder, autosomal dominant 23	*SETD5*	NM_001080517.3	c.3848_3849insC:p.(Ser1286fs)	PVS1;PS2;PM2	P	HP:0001572-Macrodontia; HP:0001249-Intellectual disability; HP:0004322-Short stature; HP:0000924-Abnormality of the skeletal system; HP:0001999-Abnormal facial shape
**GM190941**	M	Coffin-Siris syndrome 4	*SMARCA4*	NM_003072.5	c.3068A>G:p.(Glu1023Gly)	PS2; PM2; PP2; PP3	LP	HP:0006889-Intellectual disability, borderline, HP:0011968-Feeding difficulties, HP:0000708-Behavioral abnormality, HP:0000736-Short attention span, HP:0000750-Delayed speech and language development, HP:0002353-EEG abnormality, HP:0025313-Exophoria, HP:0100702-Arachnoid cyst;HP:0011937-Hypoplastic fifth toenail , HP:0010935-Abnormality of the upper urinary tract, HP:0000768- Pectus carinatum
**GM223379**	F	Coffin-Siris syndrome 4	*SMARCA4*	NM_003072.5	c.1646G>T:p.(Arg549Leu)	PS2; PM2; PP2; PP3	LP	HP:0001249-Intellectual disability;
**GM223380**	F	Coffin-Siris syndrome 3	*SMARCB1*	NM_003073.5	c.110G>A:p.(Arg37His)	PM2; PP2; PP3: PP5	LP	HP:0001249-Intellectual disability, HP:0000238-Hydrocephalus, HP:0002273-Tetraparesis, HP:0002247-Duodenal atresia, HP:0000518-Cataract
**GM183514**	F	Cornelia de Lange syndrome 2	*SMC1A*	NM_006306.4	c.1276_1282del:p.(Arg426fs)	PVS1; PS2; PM2;	LP	HP:0001249-Intellectual disability; HP:0001250-Seizures
**130091**	M	Coffin-Siris syndrome 9	*SOX11*	NM_003108.3	c.159G>T :p.(Met53Ile)	PS2;PM1;PM2; PP2; PP3	P	Neurodevelopmental delay HP:0012758, Behavioral abnormality HP:0000708, Cleft palate HP:0000175, Absent speech HP:0001344 , Inguinal hernia HP:0000023
**131749**	F	FLHS	*SRCAP*	NM_006662.3	c.7937_7938del:p.(Val2646fs)	PVS1;PS2; PM2;PP5	P	Autistic behavior HP:0000729, Intellectual disability, mild HP:0001256, Delayed speech and language development HP:0000750, Self-injurious behavior HP:0100716, Growth delay HP:0001510, Abnormal facial shape HP:0001999
**Validation cohort: Copy Number Variants (CNVs) (25 cases)**								
**NWM-020D**	F	Mental retardation, autosomal dominant 23	*SETD5*	GRCh[38]-CNV loss	3p25.3(9091710-12334937)x1	L1A;L2A;L3C;L4E;L5F	P-2.00	HP:00001249; HP:00001252; HP:000010767;HP:00001643;HP:000040253;HP:00001162; HP:00001159;HP:000011231;HP:000011333;HP:0000337;HP:0000490;HP:0000506;HP:0000431;HP:0000368;HP:0000396;HP:0000395;HP:0000343;HP:0000325;HP:0000276;HP:0000331;HP:000010211;HP:0000494
**162391**	M	Mental retardation, autosomal dominant 23	*SETD5*	GRCh[38]-CNV loss	3p26.3(52266-10683525)x1	L1A;L2A;L3C;L4E;L5F	P-2.00	HP:0001249-Intellectual disability

**GM190395**	F	Wolf-Hirschhorn syndrome	Chr4p16.13 del	GRCh[38]-CNV loss	4p16.13(71660-6479683)x1	L1A;L2A;L3C;L4E;L5F	P-2.0	HP:0001249-Intellectual disability;
**GM200157**	F	Wolf-Hirschhorn syndrome	Chr4p16.13 del	GRCh[38]-CNV loss	4p16.13(71660-13395123)x1	L1A;L2A;L3C;L4E;L5F	P-2.0	HP:0001249-Intellectual disability;
**T223**	M	Sotos syndrome	Chr.5q35	GRCh[38]-CNV loss	5q35(176463495-177956831)x1	L1A;L2A;L3C;L4E;L5F	P-2.00	HP:0100543-Cognitive impairment
**S288**	M	Hunter McAlpine syndrome	Chr.5q35-qter.dup	GRCh[38]-CNV gain	5q35(176412680-177477797)x3	G1A;G2A;G3B; L4B;L5A	P-2.05	HP:0000047-Hypospadias;HP:0003510-Severe short stature;HP:0000252-Microcephaly;HP:0000750-Delayed speech and language development; HP:0001263-Global developmental delay
**GM201583**	F	Williams-Beuren synrdome	Chr7q11.23 del	GRCh[38]-CNV loss	7q11.23(73312582-74924037)x1	L1A;L2A;L3C;L4E;L5F	P-2.0	HP:0001627-Abnormal heart morphology;
**GM192375**	M	Suspected Williams-Beuren synrdome	Chr7q11.23 del	GRCh[38]-CNV loss	7q11.23(73312582-74725057)x1	L1A;L2A;L3B;L4J;L5B	VUS-0.85	HP:0001249-Intellectual disability;
**GM193789**	F	Chr7q11.23 duplication syndrome	Chr7q11.23 dup	GRCh[38]-CNV gain	7q11.23(73312582-74725057)x3	G1A;G2A;G3A; L4E;L5F	P-1.10	HP:0001249-Intellectual disability;
**111884**	F	Kleefstra syndrome 1	*EHMT1*	GRCh[38]-CNV loss	9q34.3(136428708-138059695)x1	L1A;L2A;L3C;L4E;L5F	P-2.00	HP:0005176-Dysplastic aortic valve;HP:0000316-Hypertelorism;HP:0010804-Tented upper lip vermilion; HP:0000179-Thick lower lip vermilion; HP:0001290-Generalized hypotonia; HP:0011451-Primary microcephaly;HP:0001263-Global developmental delay;HP:0001250-Seizure
**131568**	F	Kleefstra syndrome 1	*EHMT1*	GRCh[38]-CNV loss	9q34.3(137447506-137984409)x1	L1A;L2A;L3A; L4E;L5F	P-1.10	HP:0100543-Cognitive impairment;HP:0001249-Intellectual disability;
**161978**	M	Kleefstra syndrome	*EHMT1*	GRCh[38]-CNV loss	9q34.3(135866376-138114463)x1	L1A;L2A;L3C;L4E;L5F	P-2.00	HP:0001999-Abnormal facial shape;HP:0001249-Intellectual disability
**GM181473**	F	Kleefstra syndrome 1	*EHMT1*	GRCh[38]-CNV loss	9q34.3(137666340-138059695)x1	L1A;L2A;L3A; L4E;L5F	P-1.10	HP:0001249-Intellectual disability;HP:0001007-Hirsutism
**N821**	F	Suspected Rubinstain Taybi	*CREBBP*	GRCh[38]-CNV loss	16p13.3(3461539-3805666)x1	L1A;L2C-1;L3A;L4E;L5F	P-1.00	
**112066**	M	Velocardiofacial syndrome	Chr.22q11.21del	GRCh[38]-CNV loss	22q11.21(18932429-21086225)x1	L1A; L2A; L3C; L4A; L5H	P-2.35	HP:0100543-Cognitive impairment
**112408**	M	Velocardiofacial syndrome	Chr.22q11.21del	GRCh[38]-CNV loss	22q11.21(18932429-21086225)x1	L1A; L2A; L3C; L4A; L5H	P- 2.35	HP:0100702-Arachnoid cyst; HP:0000750-Delayed speech and language development;HP:0001263-Global developmental delay
**141583**	M	Velocardiofacial syndrome	Chr.22q11.21del	GRCh[38]-CNV loss	22q11.21(18932429-21086225)x1	L1A; L2A; L3C; L4A; L5H	P- 2.35	HP:0001249-Intellectual disability;
**160892**	M	Velocardiofacial syndrome	Chr.22q11.21del	GRCh[38]-CNV loss	22q11.21(18932429-21086225)x1	L1A; L2A; L3C; L4A; L5H	P- 2.35	HP:0002463-Language impairment

**161876**	F	Velocardiofacial syndrome	Chr.22q11.21del	GRCh[38]-CNV loss	22q11.21(18932429-21086225)x1	L1A; L2A; L3C; L4A; L5H	P- 2.35	HP:0001249-Intellectual disability; HP:0005684-Distal arthrogryposis;
**GM192617**	F	Velocardiofacial syndrome	Chr.22q11.21del	GRCh[38]-CNV loss	22q11.21(18932429-21086225)x1	L1A; L2A; L3C; L4K; L4M;L5E	P-1.75	HP:0001249-Intellectual disability;HP:0001250
**150284**	M	Velocardiofacial syndrome	Chr.22q11.21del	GRCh[38]-CNV loss	22q11.21(18932429-20324240)x1	L1A;L2A;L3C;L4E;L5H	P-2.15	HP:0001249-Intellectual disability;HP:0100753-Schizophrenia
**162620**	M	Velocardiofacial syndrome	Chr.22q11.21del	GRCh[38]-CNV loss	22q11.21(18932429-20324240)x1	L1A;L2A;L3C;L4E;L5H	P-2.15	HP:0001249-Intellectual disability; HP:0001611-Nasal speech
**142071**	F	Koolen de Vreis syndrome	*KANSL1*	GRCh[38]-CNV loss	17q21.3(45640337-46082496)x1	L1A;L2A;L3A; L4C;L5A	P-1.55	HP:0001263-Global developmental delay
**152118**	F	Koolen de Vreis syndrome	*KANSL1*	GRCh[38]-CNV loss	17q21.3(45640337-46133456)x1	L1A;L2A;L3A; L4C;L5A	P-1.55	HP:0001680-Coarctation of aorta; HP:0001629-Ventricular septal defect;HP:0001249-Intellectual disability
**GM181681**	F	Koolen de Vreis syndrome	*KANSL1*	GRCh[38]-CNV loss	17q21.3(45640337-46267672)x1	L1A;L2A;L3A; L4E;L5F	P-1.1	HP:0001249-Intellectual disability;HP:0001274-Agenesis of corpus callosum
**Validation of SNV/CNV VUS /no variant (18 cases)**								
**160708**	M	Coffin-Siris syndrome 1	*ARID1B*	NM_001374828.1	c.2480C>T:p.(Ala827Val)	PM2;PP5	VUS	HP:0000729-Autistic behavior, HP:0012758-Neurodevelopmental delay , HP:0001250-Seizure , HP:0000126-Hydronephrosis, HP:0012741-Unilateral cryptorchidism, HP:0012646-Retractile testis
**150163**	M	Coffin-Siris syndrome 1	*ARID1B*	NM_001374828.1	c.3589G>A:p.(Asp1197Asn)	PP5	VUS	HP:0000729-Autistic behavior, HP:0001263-Global developmental delay, HP:0000664-Synophrys (mild), HP:0031770 (mild)-Epicanthus palpebralis , HP:0000233-Thin vermilion border , HP:0000343-Long philtrum, HP:0000319-Smooth philtrum, HP:0000430-Underdeveloped nasal alae, HP:0000193-Bifid uvula
**NWM-116D**	M	Mental retardation, XL 93	*BRWD3*	NM_153252.5	c.1233-7_1233-3del	PM2;	VUS	HP:0001249-Intellectual disability;
**GM173400**	F	Nicolaides-Baraitser syndrome	*SMARCA2*	NM_003070.5	c.2566A>G, p.(Met856Val)	PM2; PP2; PP3	VUS	HP:0001264-Spastic diplegia;HP:0000483-Astigmatism;HP:0002714-HP:0002003-Large forehead; Downturned corners of mouth;HP:0000316-Hypertelorism;HP:0001182-Tapered fingers;HP:0004209-Clonodactyly of the 5th finger
**GM203135**	F	Phenotype not corresponding to Wiedemann Steinert	*KMT2A*	NM_001197104.2	c.5959G>A:p.(Glu1987Lys)	PM2; PP2;PP3	VUS	HP:0004313; HP:0030991; HP:0000776; HP:0000252; HP:0006872
**140556**	M	Nicolaides-Baraitser syndrome	*SMARCA2*	NM_003070.5	c.2296C>G:p.(Leu766Val)	PM1; PM2; PP2; PP3	VUS	HP:0009800-Maternal diabetes , HP:0006889-Intellectual disability, borderline, HP:0001328-Specific learning disability , HP:0010522-Dyslexia , HP:0025499-Class I obesity.

**140558**	M	Nicolaides-Baraitser syndrome	*SMARCA2*	NM_003070.5	c.2296C>G:p.(Leu766Val)	PM1; PM2; PP2; PP3	VUS	HP:0006889-Intellectual disability, borderline, HP:0001511:Intrauterine growth retardation , HP:0000750:Delayed speech and language development , HP:0007018:Attention deficit hyperactivity disorder, HP:0000708 :Behavioral abnormality, HP:0001741:Phimosis , HP:0010535 Sleep apnea
**NWM-236D**	F	Cornelia de lange-like phenotype	*NIPBL*	?	?	?	?	HP:0001249-Intellectual disability, HP:0000002-Abnormality of body height;HP:0001518-Small for gestational age;HP:0001622-Premature birth;HP:0001655-Patent foramen ovale;HP:0000664-Synophrys;HP:0000347-Micrognathia
**S890**	M	Velocardiofacial syndrome	Chr.22q11.21del	GRCh[38]-CNV loss	22q11.21(20379137-21151128)x1	L1A;L2A;L3C;L4C;L5A	P-2.45	HP:0001629-Ventricular septal defect;HP:0001363-Craniosynostosis;HP:0000176-Submucous cleft hard palate; HP:0003414-Atlantoaxial dislocation; HP:0008440-C1-C2 vertebral abnormality; HP:0002308-Chiari malformation; HP:0001263-Global developmental delay; HP:0003396-Syringomyelia
**GM203534**	F	Velocardiofacial syndrome	Chr.22q11.21del	GRCh[38]-CNV loss	22q11.21(20400132-21086225)x1	L1A;L2A;L3B;L4E;L5H	P-1.70	HP:0001249-Intellectual disability;HP:0000347-Microcnathia;HP:0030084-Clinodactyly
**140901**	F	Velocardiofacial syndrome	Chr.22q11.21del	GRCh[38]-CNV loss	22q11.21(20400132-21086225)x1	L1A;L2A;L3B;L4E;L5H	P-1.70	HP:0001249-Intellectual disability; HP:0007894-Hypopigmentation of the fundus ;Nystagmus-HP:0000639;HP:0001290-Generalized hypotonia;HP:0001388-Joint laxity;
**R641**	M	Velocardiofacial syndrome	Chr.22q11.21del	GRCh[38]-CNV loss	22q11.21(21444416-22574173)x1	L1A;L2A;L3C;L4C;L5F	P-2.00	HP:0001249-Intellectual disability;
**141494**	F	Velocardiofacial syndrome	Chr.22q11.21del	GRCh[38]-CNV loss	22q11.21(21444416-22574173)x1	L1A;L2A;L3C;L4B;L5A	P-2.50	HP:0001249-Intellectual disability; HP:0001627-Abnormal heart morphology;
**S257**	F	Velocardiofacial syndrome	Chr.22q11.21del	GRCh[38]-CNV loss	22q11.21(20721287-21025669)x1	L1A;L3A;L4C;L5A	VUS-0.55	HP:0001249-Intellectual disability; HP:0000104-Renal agenesis;HP:0007874-Almond-shaped palpebral fissure;HP:0001363-Craniosynostosis; HP:0010823-Ridged cranial sutures;HP:0002553-Highly arched eyebrow;HP:0001252-Hypotonia;HP:0000347-Micrognathia; HP:0011451-Primary microcephaly; HP:0002079-Hypoplasia of the corpus callosum;
**131777**	M	Velocardiofacial syndrome	Chr.22q11.21del	GRCh[38]-CNV loss	22q11.22(21968733-22215491)x1	L1A;L3A;L4E;L5F	VUS-0.10	HP:0007429-Few cafe-au-lait spots;HP:0009719-Hypomelanotic macule;HP:0000729-Autistic behavior
**GM194370**	M	Velocardiofacial syndrome	Chr.22q11.21del	GRCh[38]-CNV loss	22q11.22(21968733-22215491)x1	L1A;L2B;L3A;L4C;L5F	VUS-0.10	HP:0007272-Progressive psychomotor deterioration;
**GM193223**	M	Velocardiofacial syndrome	Chr.22q11.21del	GRCh[38]-CNV loss	22q11.22(21968733-22215491)x1	L1A;L3A;L4J;L5B	VUS-(-0.60)	HP:0000717-Autism

**GM191544**	M	Velocardiofacial syndrome	Chr.22q11.21del	GRCh[38]-CNV loss	22q11.22(22655814-23285204)x1	L1A;L2B;L3C;L4J;L5E	VUS-0.30	HP:0002355-Difficulty walking;HP:0001263-Global developmental delay;
**GM193550**	M	Velocardiofacial syndrome	Chr.22q11.21del	GRCh[38]-CNV loss	22q11.22(22655814-23285204)x1	L1A;L2B;L3C;L4J;L5E	VUS-0.30	HP:0007018-Attention deficit hyperactivity disorder; HP:0001268-Mental deterioration
**XCI cases screening (20)**								
**NWM-021D**	F	Syndromic intellectual disability	/	/	/	/	/	HP:0001249-Intellectual disability; HP:0000717-Autism;HP:0001257-Spasticity; HP:0001347-Hyperreflexia;HP:0009487-Ulnar deviation of the hand;HP:0100702-Arachnoid cyst;HP:0002280-Enlarged cisterna magna;HP:0000383-Abnormality of periauricular region;HP:0000372-Abnormality of the auditory canal;HP:0000413-Atresia of the external auditory canal;HP:0000581-Blepharophimosis;HP:0000508-Ptosis;HP:0005280-Depressed nasal bridge;HP:0000537-Epicanthus inversus
**141078**	M	XCI skeweing	/	/				
**162199**	M	XCI skeweing	/	/				
**150692**	M	XCI skeweing	/	/				
**140041**	M	XCI skeweing	/	/				
**160035**	M	XCI skeweing	/	/				
**152994**	F	XCI skeweing	/	/				
**141345**	F	XCI skeweing	/	/				
**210581**	F	XCI skeweing	/	/				
**150689**	F	XCI skeweing	/	/				
**170809**	F	XCI skeweing	/	/				
**29D**	F	XCI skewing	/	/				
**6D**	F	XCI skewing	/	/				
**173D**	F	XCI skewing	/	/				
**164D**	M	XCI skewing	/	/				
**FM0711016_92**	M	XCI skewing	/	/				
**90D**	M	XCI skewing	/	/				
**43D**	M	XCI skewing	/	/				
**22D**	M	XCI skewing	/	/				
**111092**	M	ATRX-like phenotype	*ATRX*	NM_000489.6	c.134-4884_242+41del	L1A;L2E;L3A;L5D	P-1.20	HP:0010864-Intellectual disability, severe, HP:0000286/Epicanthus , HP:0010806/U-Shaped upper lip vermilion, HP:0000194-Open mouth, HP:0001883-Talipes, HP:0002307-Drooling, HP:0001270-Motor delay , HP:0001344-Absent speech, HP:0012736-Profound global developmental delay

Supplemental Table 2: filtered genome sequencing variants for cases 150163 and 218D (see link- https://www.medrxiv.org/content/10.1101/2022.09.18.22277970v1 (patient 4722))

NIPBL **(NM_133433.4)**	**effect**	**GnomAD**	**Inheritance**
c.-80+35690G>A (intron 1/46)	no effect?	not reported	paternal
c.1495+3191A>G (intron 9/46)	New donor splice site: Activation of a cryptic donor site.	not reported	paternal
c.7861-1201G>C (intron 45/46)	Alteration of auxiliary sequence: Significant alteration if ESE/ESS motifs ration	not reported	paternal

Supplemental table 3: ***SMARCA2*** **tested variants**

Variant	Category
NM_001289396.1(SMARCA2):c.1477_1479del, p.(Lys493del)	BAFopathy
NM_001289396.1(SMARCA2):c.2255G>C, p.(Gly752Ala)	BAFopathy
NM_001289396.1(SMARCA2):c.2261G>C, p.(Gly754Ala)	BAFopathy
NM_001289396.1(SMARCA2):c.2264A>G, p.(Lys755Arg)	BAFopathy
NM_001289396.1(SMARCA2):c.2348C>G, p.(Ser783Trp)	BAFopathy
NM_001289396.1(SMARCA2):c.2486C>T, p.(Thr829Ile)	BAFopathy
NM_001289396.1(SMARCA2):c.2558G>T, p.(Gly853Val)	BAFopathy
**NM_001289396.1(SMARCA2):c.2564G>C, p.(Arg855Pro)**	**BAFopathy**
NM_001289396.1(SMARCA2):c.2639C>T, p.(Thr880Ile)	BAFopathy
NM_001289396.1(SMARCA2):c.2642G>T, p.(Gly881Val)	BAFopathy
NM_001289396.1(SMARCA2):c.2647C>G, p.(Pro883Ala)	BAFopathy
NM_001289396.1(SMARCA2):c.2648C>T, p.(Pro883Leu)	BAFopathy
NM_001289396.1(SMARCA2):c.2671C>T, p.(Leu891Phe)	BAFopathy
NM_001289396.1(SMARCA2):c.2744C>A, p.(Ala915Asp)	BAFopathy
NM_001289396.1(SMARCA2):c.3209T>A, p.(Leu1070Gln)	BAFopathy
NM_001289396.1(SMARCA2):c.3313C>A, p.(Arg1105Ser)	BAFopathy
NM_001289396.1(SMARCA2):c.3404T>C, p.(Leu1135Pro)	BAFopathy
NM_001289396.1(SMARCA2):c.3464A>C, p.(Gln1155Pro)	BAFopathy
NM_001289396.1(SMARCA2):c.3475C>G, p.(Arg1159Gly)	BAFopathy
NM_001289396.1(SMARCA2):c.3476G>T, p.(Arg1159Leu)	BAFopathy
NM_001289396.1(SMARCA2):c.3485G>A, p.(Arg1162His)	BAFopathy
NM_001289396.1(SMARCA2):c.3493C>A, p.(Gln1165Lys)	BAFopathy
NM_001289396.1(SMARCA2):c.3573G>C, p.(Lys1191Asn)	BAFopathy
NM_001289396.1(SMARCA2):c.3602C>T, p.(Ala1201Val)	BAFopathy
NM_001289396.1(SMARCA2):c.3623C>G, p.(Ser1208Cys)	BAFopathy
NM_001289396.1(SMARCA2):c.3849G>T, p.(Trp1283Cys)	BAFopathy
NM_001289396.1(SMARCA2):c.1458C>G, p.(Asn486Lys)	BIS
NM_001289396.1(SMARCA2):c.1534G>A, p.(Glu512Lys)	BIS
NM_001289396.1(SMARCA2):c.1538G>T, p.(Gly513Val)	BIS
NM_001289396.1(SMARCA2):c.1573C>T, p.(Arg525Cys)	BIS
NM_001289396.1(SMARCA2):c.1574G>A, p.(Arg525His)	BIS
NM_001289396.1(SMARCA2):c.1585C>G, p.(Leu529Val)	BIS
**NM_001289396.1(SMARCA2):c.2566A>G, p.(Met856Val)**	**BIS**
NM_001289396.1(SMARCA2):c.2725T>A, p.(Phe909Ile)	BIS
NM_001289396.1(SMARCA2):c.2809C>T, p.(Arg937Cys)	BIS
NM_001289396.1(SMARCA2):c.2810G>A, p.(Arg937His)	BIS

Supplemental Materials and methods

X chromosome inactivation (XCI) analysis

XCI was tested in blood extracted DNA using an in-house developed protocol, as previously described.[1] In short, the XCI pattern was calculated using three microsatellite polymorphic markers to avoid uninformative results: (i) the CA-repeat in the promoter region of the SLIT and NTRK Like Family Member 4 *(SLITRK4)* gene; (ii) the CAG-repeat located in exon 1 of androgen receptor (*AR*) gene; (iii) the CA and AG tandem repeats in the first intron of Proprotein Convertase Subtilisin/Kexin Type 1 Inhibitor (*PCSK1N*) gene.

Genome sequencing analysis for case 150163

Genome sequencing was outsourced to BGI (Sequencing Platform: DNBseq; Sequencing read Length: PE100). After sequencing, raw data with adapter sequences or low-quality sequences were filtered using the SOAPnuke software (filter parameters: " -n 0.001 -l 10 --adaMR 0.25 --minReadLen 100"). We obtained 540,292,479 clean reads for a total of 108,058,495,800 bases. Q20: 98.56; Q30: 94.75.

Raw sequences were processed and analyzed using an in-house implemented pipeline previously described[2,3] which is based on the GATK Best Practices.[4] Briefly, in the pre-processing step reads were aligned to the GRCh38 genome assembly using BWA-MEM,[5] duplicates were marked with samtools,[6] markdup (v1.16) and base quality scores recalibrated with GATK[4] (v4.2.1) BaseRecalibrator and ApplyBQSR. Single Nucleotide Variants (SNVs) and insertions and deletions <50 bp were called using GATK HaplotypeCaller and GenotypeGVCFs. We used Ensembl VEP v.104[7] and dbNSFP v.4.0[7] tools for variants functional annotation, including Combined Annotation Dependent Depletion (CADD) v.1.3, [8] Mendelian Clinically Applicable Pathogenicity (M-CAP) v.1.0[9] and Intervar v.0.1.6 for functional impact prediction. [10]

Thereby, the analysis was narrowed to variants which affect coding sequences or splice site regions. Moreover, high-quality variants were filtered against public databases (dbSNP150 and GnomAD ver.2.0.1) so that only variants with unknown frequency or having MAF <0.1%, as well as variants occurring with frequency < 1% in our population-matched database (~2000 exomes) were considered. Structural Variations (SVs) were called using Manta v1.6.0,[8] Delly v1.1.6,[9] SvABA v1.1.0,[10] and LUMPY v0.3.1,[11] and individual results were combined in a single VCF file using a home-made script. The resulting VCF file was annotated using AnnotSV v3.1.3,[12] and subsequentially filtered by removing SVs found in population databases with a frequency > 1% or in the ENCODE blacklist.

We carefully verified the presence of rare variants in the genomic region of the five known CdLS genes (*NIPBL, SMC1A, SMC3, RAD21, HDAC8*).

Supplemental References

1. Giovenino C, Trajkova S, Pavinato L, et al. Skewed X-chromosome inactivation in unsolved neurodevelopmental disease cases can guide re-evaluation For X-linked genes. Eur J Hum Genet. 2023.
2. Bauer CK, Calligari P, Radio FC, et al. Mutations in KCNK4 that Affect Gating Cause a Recognizable Neurodevelopmental Syndrome. Am J Hum Genet. 2018;103(4):621-630.
3. Flex E, Martinelli S, Van Dijck A, et al. Aberrant Function of the C-Terminal Tail of HIST1H1E Accelerates Cellular Senescence and Causes Premature Aging. Am J Hum Genet. 2019;105(3):493-508.
4. Van der Auwera GA, Carneiro MO, Hartl C, et al. From FastQ data to high confidence variant calls: the Genome Analysis Toolkit best practices pipeline. Curr Protoc Bioinformatics. 2013;43:11 10 11-33.
5. Li H, Durbin R. Fast and accurate long-read alignment with Burrows-Wheeler transform. Bioinformatics. 2010;26(5):589-595.
6. Danecek P, Bonfield JK, Liddle J, et al. Twelve years of SAMtools and BCFtools. Gigascience. 2021;10(2).
7. McLaren W, Gil L, Hunt SE, et al. The Ensembl Variant Effect Predictor. Genome Biol. 2016;17(1):122.
8. Chen X, Schulz-Trieglaff O, Shaw R, et al. Manta: rapid detection of structural variants and indels for germline and cancer sequencing applications. Bioinformatics. 2016;32(8):1220-1222.
9. Rausch T, Zichner T, Schlattl A, Stütz AM, Benes V, Korbel JO. DELLY: structural variant discovery by integrated paired-end and split-read analysis. Bioinformatics. 2012;28(18):i333-i339.
10. Wala JA, Bandopadhayay P, Greenwald NF, et al. SvABA: genome-wide detection of structural variants and indels by local assembly. Genome Res. 2018;28(4):581-591.
11. Layer RM, Chiang C, Quinlan AR, Hall IM. LUMPY: a probabilistic framework for structural variant discovery. Genome Biol. 2014;15(6):R84.
12. Geoffroy V, Herenger Y, Kress A, et al. AnnotSV: an integrated tool for structural variations annotation. Bioinformatics. 2018;34(20):3572-3574.
13. Gerber CB, Fliedner A, Bartsch O, et al. Further characterization of Borjeson-Forssman-Lehmann syndrome in females due to de novo variants in PHF6. Clin Genet. 2022;102(3):182-190.
14. Allen MD, Freund SM, Zinzalla G, Bycroft M. The SWI/SNF Subunit INI1 Contains an N-Terminal Winged Helix DNA Binding Domain that Is a Target for Mutations in Schwannomatosis. Structure. 2015;23(7):1344-1349.
